# Polymeric Network Hierarchically Organized on Carbon Nano-onions: Block Polymerization as a Tool for the Controlled Formation of Specific Pore Diameters

**DOI:** 10.1021/acsapm.1c01788

**Published:** 2022-03-17

**Authors:** Gabriela Siemiaszko, Agnieszka Hryniewicka, Joanna Breczko, Olivia Fernandez Delgado, Karolina H. Markiewicz, Luis Echegoyen, Marta E. Plonska-Brzezinska

**Affiliations:** † Department of Organic Chemistry, Faculty of Pharmacy with the Division of Laboratory Medicine, Medical University of Bialystok, Mickiewicza 2A, 15-222 Bialystok, Poland; ‡ Faculty of Chemistry, University of Bialystok, Ciolkowskiego 1K, 15-245 Bialystok, Poland; § Department of Chemistry, University of Texas at El Paso, 500 West University Avenu, El Paso, Texas 79968 United States

**Keywords:** carbon nano-onion, carbon nanostructure, porous material, RAFT polymerization, supercapacitor

## Abstract

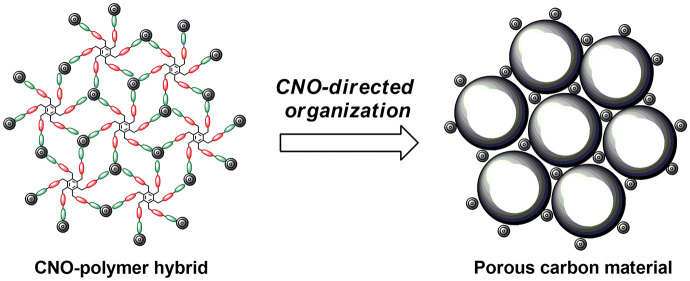

The organization of specific pores in carbonaceous three-dimensional networks is crucial for efficient electrocatalytic processes and electrochemical performance. Therefore, the synthesis of porous materials with ordered and well-defined pores is required in this field. The incorporation of carbon nanostructures into polymers can create material structures that are more ordered in comparison to those of the pristine polymers. In this study we applied polymer-templated methods of carbon material preparation, in which outer blocks of the star copolymers form the carbon skeleton, while the core part is pore-forming. Well-defined 6-*star*-(poly(methyl acrylate)-*b*-poly(4-acetoxystyrene)) dendrimers were synthesized by reversible addition–fragmentation chain-transfer polymerization. They were then transformed into poly(4-vinylphenol) derivatives (namely 6-*star*-(poly(methyl acrylate)-*b*-poly(4-vinylphenol)), subjected to polycondensation with formaldehyde, and pyrolyzed at 800 °C. Cross-linking of phenolic groups provides a polymer network that does not depolymerize by pyrolysis, unlike poly(methyl acrylate) chains. The selected star polymers were attached to carbon nano-onions (CNOs) to improve the organization of the polymer chains. Herein, the physicochemical properties of CNO-polymer hybrids, including the textural and the electrochemical properties, were compared with those of the pristine pyrolyzed polymers obtained under analogous experimental conditions. For these purposes, we used several experimental and theoretical methods, such as infrared, Raman, and X-ray photoelectron spectroscopy, nitrogen adsorption/desorption measurements, scanning and transmission electron microscopy, and electrochemical studies, including cyclic voltammetry. All of the porous materials were evaluated for use as supercapacitors.

## 1 Introduction

It is key to use carbon nanostructures (CNs) in the design of porous materials to achieve high-quality modification of the three-dimensional (3D) architecture and organization of the porous structure in such a way that the materials obtained possess an orderly pore distribution and a homogeneous pore size distribution (micro, meso, and macro). The presence of micropores in porous materials is a result of the surface properties of the nanoparticles and their size. Larger pores, such as meso- and macropores, arise mainly from cross-linking of the oligomeric/polymeric chains in the presence of CNs. The incorporation of functionalized CNs leads to organized polymerization in a 3D manner. On the molecular level, the forces that will have the main influence on the organization of the polymeric network are (i) the surface chemistry of the CNs (the type and number of functional groups on the surface of the CNs, which determines where polymerization will occur), (ii) hydrogen bonding as a result of the chemical nature of the polymers, and (iii) the experimental conditions applied during their formation. Different organizations of the polymeric network result in different physicochemical properties and functionalities. In this respect, controlling the structure of the framework by controlling the organization of the pores and their sizes is required for further applications of these materials in many fields, where one of the critical parameters is their porosity.^1^ The organization of specific pores is crucial for electrocatalytic processes and electrochemical performance. Well-controlled micro- and mesoporous carbon structures are attractive electrode materials for supercapacitors (SCs) due to their high specific surface area, large pore sizes and volumes, simple and nontoxic synthesis, high chemical stability, and improved shelf-life-based cycling efficiency.^2,3^ Ordered pores improve the capacitive properties of materials by reducing the distance of ion diffusion from the bulk electrolyte into the micropores, as they serve as buffering reservoirs for ions and their transport pathways.^4^

The synthesis of well-controlled mesoporous carbon materials is usually accomplished by hard- or soft-templating methods.^3^ Hard-templating requires synthesis of the carbon-yielding component in the presence of impregnated presynthesized hard templates (i.e., silica), followed by carbonization and template removal.^5^ The soft-templating process applies the self-assembly of pore-forming amphiphilic compounds, such as surfactants (i.e., Pluronics) in the presence of the carbon-source component or block copolymers.^6^ These methods, however, have some limitations, such as the removal of inorganic templates, which require the use of toxic and corrosive substances, or the difficult and precise preparation of soft templates.^7^ Additionally, to increase the porosity of materials, physical activation methods using oxidizing gases (e.g., CO_2_) or activating chemicals (e.g., KOH) are used.^8^ However, despite the increased specific surface area of the material, this physical activation method may result in an irregular distribution of the resulting micropores and thus limited access of the electrolyte to the pore surface, resulting in a reduced electrochemical performance at high current densities.

Nanostructured hybrid materials used for energy conversion and storage employ block copolymers to control their morphology due to the copolymer soft-templating method.^9^ For example, controllable mesoporous carbons can be obtained by a method consisting of self-assembly, thermal stabilization (at 200–300 °C), and pyrolysis of certain diblock polymers containing polyacrylonitrile (PAN) as a carbon-yielding polymer block, which results in the formation of N-doped nanographene with a tunable structure depending on the molecular design of its precursors.^10^ The second polymer component, the so-called sacrificial block, e.g., poly(butyl acrylate) or poly(methyl methacrylate), undergoes depolymerization during pyrolysis, ensuring the formation of pores in the carbon skeleton.^11^ Strict control over the morphology of polymer chains can be achieved by controlled radical polymerization methods, reversible addition–fragmentation chain transfer (RAFT) polymerization, or atom transfer radical polymerization (ATRP).^12−15^ The RAFT method has an advantage over ATRP because it does not require the use of transition-metal complexes that could contaminate the product and distort the electrochemical performance. Known morphologies of polymers transformed into porous carbon materials include, apart from block polymers, molecular brushes^14^ and hairy nanoparticles.^15^ To the best of our knowledge, using star polymers as the precursors for the synthesis of porous carbon materials is an approach not yet described in the literature.

Phenol– or resorcinol–formaldehyde polycondensation is a known method for obtaining polymers transformed into porous nanocarbons. Examples include the use of soft or hard templating, as well as the application of both.^16^ Some of the porous nanocarbons were tested for electrochemical properties and exhibited high capacitance values, 100–200 F g^–1^ in an aqueous medium and 50–150 F g^–1^ in an organic medium.^17^ For example, nonactivated carbon, thus possessing a narrower pore-size distribution, exhibits a maximum capacitance of 175.9 F g^–1^ in 6 mol dm^–3^ KOH at a scan rate of 1 mV s^–1^ using a three-electrode system (vs Ag/AgCl).^18^ Moreover, the Brunauer–Emmett–Teller (BET) surface area of the sample was 473 m^2^ g^–1^, and the total pore volume was 0.287 cm^3^ g^–1^.

Herein, the synthesis of porous carbon materials using star polymers and carbon nano-onions (CNOs) as a nanostructural matrix for controlled polymerization is presented. CNOs are multilayered fullerenes obtained mainly by thermal annealing of nanodiamonds (NDs) at high temperatures under high vacuum.^19^ CNOs are highly conductive, thermally stable, corrosion-resistant, and high surface to volume ratio materials attractive for applications as electrodes in electric double-layer capacitors (EDLCs).^20^ Due to the low energy density and capacity of these nanomaterials, many research groups have attempted to modify CNOs to improve their electrochemical properties and exploit them in the production of supercapacitors.^2,21^ These attempts involve doping with heteroatoms (i.e., sulfur),^22^ addition of a metal oxide active in redox reactions,^23^ and covalent functionalization with organic compounds.^24^ Importantly, resorcinol–formaldehyde resins with Pluronic as a soft template and CNO addition were prepared, followed by pyrolysis, while their capacitance and resistance were tailored by changing the ratio of CNs to resin.^25^

In this study, 6-*star*-(poly(methyl acrylate)-*b*-poly(4-acetoxystyrene)) dendrimers, **6-***star-***(PMA-***b***-PAS)**, were synthesized by RAFT polymerization using a new chain transfer agent (CTA). Subsequently, they were transformed into 6-*star*-(poly(methyl acrylate)-*b*-poly(4-vinylphenol)), **6-***star-***(PMA-***b***-PVPh)**, and condensed with formaldehyde. Interestingly, the carbon-yielding application of poly(4-vinylphenol) blocks cross-linked using HCHO has not been reported thus far. The selected star polymers were attached to CNOs to improve the organization of polymer chains, resulting in higher porosity and better electrochemical performance of the resulting pyrolyzed hybrids. The physicochemical properties of all materials, including the structure, porosity, and electrochemical performance, were investigated.

## 2 Experimental Section

### 2.1 Materials

Commercially available nanodiamond (ND) powder with a crystal size of between 4 and 6 nm (Carbodeon μDiamondMolto and ND content greater than 97 wt %) was used for the preparation of CNOs (an annealing treatment under an inert atmosphere and reduced pressure of ultradispersed ND particles).^19^*N,N-*Dimethylformamide (DMF, POCH S.A., Poland) was distilled over phosphorus pentoxide (P_2_O_5_, pure, Honeywell, USA), tetrahydrofuran (THF, Supelco, USA) was distilled with sodium (Honeywell, USA), and benzophenone (99%, Aldrich, Germany). DMF, THF, dichloromethane (DCM, Honeywell, USA), and acetone (Stanlab, Poland) were dried over 4 Å molecular sieves (POCH S.A., Poland) before use. 2,2′-Azobis(2-methylpropionitrile) (AIBN, ≥95%, POL-AUR, Poland) and potassium ethyl xanthogenate (KSCSOEt, 96%, Aldrich, Germany) were recrystallized from methanol (MeOH, Chempur, Poland) or ethanol (EtOH, POCH S.A., Poland), respectively, before use. Hexakis(bromomethyl)benzene (98%, Aldrich, Germany), 2-bromopropionyl bromide (97%, Aldrich, Germany), MeOH, EtOH, and hexanes (Stanlab, Poland), ethyl acetate (AcOEt, POCH S.A., Poland), potassium hydroxide (KOH, POCH S.A., Poland), sodium hydroxide (NaOH, 97%, Aldrich, Germany), hydrochloric acid (HCl, 35–38%, Chempur, Poland), formaldehyde (HCHO, 37 wt % in H_2_O, Aldrich, Germany), magnesium sulfate (MgSO_4_, Chempur, Poland), triblock poly(ethylene oxide)-*b*-poly(propylene oxide)-*b*-poly(ethylene oxide) copolymer Pluronic F-127 (PEO_106_-PPO_70_-PEO_106_, *M*_w_ = 12 600 g mol^–1^, pure, Aldrich, Germany), and silica gel (0.040–0.063 mm, Merck, Germany) were used as received. Water was distilled using a DE 10 Plus distiller. Methyl acrylate (99%, Aldrich, Germany) and 4-acetoxystyrene (97%, FluoroChem, United Kingdom) were filtered through neutral alumina (Merck, Germany) before use. Ethanesulfonyl azide (EtSO_2_N_3_) was synthesized according to the literature procedure using ethanesulfonyl chloride (≥95%, Aldrich, Germany) and sodium azide (pure, Aldrich, Germany).^26^ The glassware and potassium bromide (KBr, ≥99%, Aldrich, Germany) were dried in a furnace at 120 °C overnight before use. Deuterated solvents, chloroform-*d* (CDCl_3_) and dimethyl sulfoxide (DMSO-*d*_*6*_), were purchased from Euroisotop (United Kingdom). The following chemicals were used for electrochemical measurements: acetonitrile (ACN, anhydrous, 99.8%, Sigma-Aldrich, Germany), tetrabutylammonium hexafluorophosphate (TBAPF_6_, ≥99.0%, Fluka Analytical, Switzerland), tetrabutylammonium perchlorate (TBAP, ≥99.0%, Fluka Analytical, Switzerland), tetrabutylammonium acetate (TBA acetate, ≥99.0%, Sigma-Aldrich, Switzerland), tetrabutylammonium tetrafluoroborate (TBABF_4_, ≥99.0%, Sigma-Aldrich, Switzerland), and tetraethylammonium hexafluorophosphate (TEAPF_6_, ≥99.0%, Sigma-Aldrich, Switzerland).

### 2.2 Methods

High-resolution transmission electron microscopy (HRTEM) was performed using a Titan G2 HRTEM microscope (FEI Company) equipped with a field emission gun (FEG). The electron beam accelerating voltage was 300 kV. HRTEM imaging of the sample microstructure was performed in bright-field mode using a CCD camera as a detector. The scanning electron microscope (SEM) measurements were performed using an INSPECT S50 microscope (FEI, Japan). The accelerating voltage of the electron beam was 15 keV. Before the measurements, a gold layer with a thickness of 7 nm was sputtered on the surface of the analyzed samples.

X-ray photoelectron spectroscopy (XPS) was performed using an ultrahigh-vacuum chamber (PREVAC) with a base pressure of below 10^–8^ mbar using an Al Kα nonmonochromatic radiation source (1486.7 eV, 12 kV, 12 mA; VG Scienta SAX 100) and monochromator (VG Scienta XM 780). Detection of emitted photoelectrons was performed using a Scienta R4000 hemispherical analyzer. A low-resolution survey run (0–1200 eV) at a pass energy of 200 eV was carried out. The C 1s, O 1s, and N 1s high-resolution spectra were recorded at a pass energy of 50 eV at room temperature. All of the spectra were fitted by Shirley background subtraction prior to Gaussian–Lorentzian functions using CasaXPS software (Casa Software Ltd.).

The room-temperature Raman spectra were taken with a Renishaw inVia confocal spectrometer (United Kingdom). The parameters used for the Raman measurements were as follows: laser with a wavelength of 785 nm (2.33 eV), power of the laser beam of 2 mW, and a spectral resolution of 2 cm^–1^. The spectra obtained after normalization were analyzed using OMNIC spectroscopy software. Fourier transform infrared spectroscopy (FTIR) was performed using a Thermo Scientific Nicolet IN10 MX microscope (USA). The spectra were recorded with a KBr pellet using a microscope in transmission mode. The spectra were collected at a resolution of 4 cm^–1^, and 64 scans were averaged to obtain a single spectrum. ^1^H and ^13^C NMR spectra were recorded on an Agilent VNMRS system operated at 500 MHz for ^1^H NMR and 126 MHz for ^13^C NMR. Chemical shifts δ are given in ppm, referenced to the solvent peak of CDCl_3_, defined at δ 7.26 (^1^H NMR) or δ 77.16 (^13^C NMR), or DMSO-*d*_6_, defined at δ 2.50 (^1^H NMR). The following abbreviations were used for multiplicities: s (singlet), d (doublet), t (triplet), q (quartet), m (multiplet).

Selected materials were characterized by thermogravimetric analysis (TGA) using a Mettler Toledo Star TGA/DSC unit. A sample weighing 2–3 mg was placed in an aluminum oxide crucible and heated from 50 to 900 °C. A heating rate of 10 °C min^–1^ and an Ar flow rate of 40 mL min^–1^ were used. An empty pan was used as a reference. Nitrogen adsorption/desorption measurements were performed at −196 °C using an ASAP 2020 analyzer (Micromeritics Corp., USA). Before the tests were started, the samples were outgassed for 4 h at 120 °C under reduced pressure. Size exclusion chromatography (SEC) was performed using a high-performance liquid chromatograph (Merck-Hitachi, Germany) equipped with a Phenogel Linear chromatography column, 5 μm, 300 mm × 7.8 mm (Merck, Germany), and a PL-ELS 1000 light scattering detector. A mixture of narrowly dispersed polystyrene molecular weight standards (1000000–500 Da) was used to calibrate the molecular weight distribution. The sample was analyzed at a concentration of 10 mg mL^–1^ in THF. The separation process was carried out at 33 °C with a mobile phase volume flow (THF) of 1.5 mL min^–1^. Nitrogen was used as the nebulizer gas at a flow rate of 2.0 L min^–1^ and a spray temperature of 110 °C. The volume of the dosed sample was 10 μL.

Samples were pyrolyzed using a Carbolite Gero STF 16/180 + 3216 Controller tube furnace. All of the PAS-derived materials were heated to 800 °C with a ramp rate equal to 10 °C min^–1^ and pyrolyzed for 3 h at 800 °C under a constant Ar flow. Next, the samples were cooled to RT with a ramp rate equal to 10 °C min^–1^ under a constant Ar flow. Voltammetric studies were performed using a PGSTAT 302N potentiostat (Autolab B.V., Metrohm, Utrecht, The Netherlands) with a three-electrode system [glassy carbon (GCE, Ø 2 mm) as the working electrode, Ag/AgCl as the reference electrode, and Pt mesh (0.25 mm^2^) as the counter electrode]. Before the measurements, the surface of the GCE was polished with carborundum paper and modified by using 15 μL of the synthesized material solution (3 mg mL^–1^ in EtOH) with the addition of conductive carbon paint (CP, SPI Supplies, USA). Then the solvent was evaporated at RT under an Ar atmosphere. All measurements were carried out in ACN solution containing 0.1 M supporting electrolyte (TBAPF_6_, TBAP, TBA acetate, TBABF_4_, or TEAPF_6_).

### 2.3 Synthetic Procedures

The synthesis of hexakis(*S*-methyl-*O*-ethyl dithiocarbonate)benzene (CTA), general procedure for the synthesis of **6-***star***-(PMA)** polymers (Table S1), the synthesis of a 2-bromopropionyl CNO derivative (**CNO-Br**), and the synthesis of a dithiocarbonate CNO derivative (**CNO-SC(S)OEt**) are reported in the Supporting Information.

#### 2.3.1 General Procedure for the Synthesis of **6-***star***-(PMA-*b*-PAS)** Polymers

The **6-***star***-(PMA)** polymer (see the Supporting Information), 4-acetoxystyrene, and the first of two equal portions of AIBN were dissolved in DMF. Ar was bubbled through this solution for 15 min. The reaction mixture was stirred for 48 h at 70 °C under Ar. The second portion of AIBN was added after 24 h. The polymers were then precipitated with MeOH, filtered, dried on tissue paper, and vacuum-dried, affording the products as white solids (Table S2). **6-***star***-(PMA**_**25**_**-***b***-PAS**_**200**_**)**: ^1^H NMR (DMSO-*d*_*6*_, 500 MHz) characteristic signals δ 7.00–6.30 (m, −C_6_*H*_4_−), 4.63 (m, −OC*H*_2_−), 3.58 (s, −OC*H*_3_), 2.25–2.15 (m, −CH_2_C*H*C(O)–, −C(O)C*H*_3_), 1.85–1.15 (m, C*H*_2_CHC(O)–, −C*H*_2_C*H*C_6_H_4_−).

#### 2.3.2 General Procedure for the Preparation of **6-***star*-**(PMA-*b*-PVPh)** Polymers

The **6-***star***-(PMA-***b***-PAS)** polymer was suspended in MeOH. Then a 0.5 M solution of KOH in MeOH was added, and the reaction mixture was stirred for 16 h at 80 °C. The reaction mixture was then cooled to RT, and the product was precipitated with 1 M HCl, followed by rinsing with distilled H_2_O and oven-drying at 80 °C. The resulting products were pale orange powders (Table S3). **6-***star***-(PMA**_**25**_**-***b***-PVPh**_**200**_**)**: ^1^H NMR (DMSO-*d*_*6*_, 500 MHz) characteristic signals δ 8.95 (s, −OH), 6.70–6.10 (m, −C_6_*H*_4_−), 3.57 (s, −OC*H*_3_), 2.25–2.15 (m, −CH_2_C*H*C(O)−), 2.00–0.90 (m, C*H*_2_CHC(O)–, −C*H*_2_C*H*C_6_H_4_−).

#### 2.3.3 General Procedure for the Polycondensation of **6-***star***-(PMA-*b*-PVPh)** Polymers with HCHO(CP-(1–4))

The **6-***star***-(PMA-***b***-PVPh)** polymer was suspended in 10% NaOH. A solution of HCHO (first of two portions) was added dropwise, and the reaction mixture was stirred at 90 °C for 5 h. The second portion of HCHO was then added, and stirring was continued for 15 h at 90 °C. The precipitate was then filtered, rinsed with H_2_O, and oven-dried at 100 °C overnight, affording the product as a green-brown solid. Synthetic details are given in Table S4.

#### 2.3.4 General Procedure for the Synthesis of **6-***star*-**(PMA-*b*-PAS-N_3_)** Polymers

The **6-***star*-**(PMA–*b*-PAS)** polymer, EtSO_2_N_3_, and AIBN (equal portion added every 1 h, total 6 h) were dissolved in anhydrous DMF, and the reaction mixture was stirred for 24 h at 70 °C under Ar. The polymer was then precipitated with MeOH, followed by filtration and vacuum drying, affording the product as a white powder (Table S5). **6-***star***-(PMA**_**25**_**-***b***-PAS**_**200**_**-N**_**3**_**)**: ^1^H NMR (DMSO-*d*_*6*_, 500 MHz) characteristic signals δ 7.00–6.25 (m, −C_6_*H*_4_−), 3.57 (s, −OC*H*_3_), 2.30–2.10 (m, −CH_2_C*H*C(O)–, −OC(O)C*H*_3_), 1.85–1.15 (m, C*H*_2_CHC(O)–, −C*H*_2_C*H*C_6_H_4_−).

#### 2.3.5 General Procedure for the Synthesis of **6-***star***-(PMA-*b*-PAS)-CNO** Hybrids

The **6-***star***-(PMA-***b***-PAS-N**_**3**_**)** polymer and CNOs in anhydrous DMF (10 mL) were stirred for 18 h at 130 °C under Ar. The products were precipitated with MeOH, followed by vacuum drying, affording dark gray to black powders. Synthetic details are given in Table S6.

#### 2.3.6 General Procedure for the Preparation of **6-***star***-(PMA-*b*-PVPh)-CNO** Hybrids

The **6-star-(PMA-*b*-PAS)-CNO** hybrid was suspended in MeOH. Then a 0.5 M solution of KOH in MeOH was added, and the reaction mixture was stirred for 16 h at 80 °C. The reaction mixture was then cooled, and the product was precipitated with 1 M HCl, followed by rinsing with distilled H_2_O and oven-drying at 80 °C. The resulting products were black powders. Synthetic details are given in Table S7.

#### 2.3.7 General Procedure for the Polycondensation of **6-**star**-(PMA-*b*-PVPh)-CNO** Hybrids with HCHO**(CP-(5-6))**

**The 6-***star***-(PMA-***b***-PVPh)-CNO** hybrid was suspended in 10% NaOH. A solution of HCHO (first of two portions) was added dropwise, and the reaction mixture was stirred at 90 °C for 5 h. The second portion of HCHO was then added, and stirring was continued for the next 15 h at 90 °C. The black precipitate was then filtered, rinsed with H_2_O, and oven-dried at 100 °C overnight, affording the product as a black solid. Synthetic details are given in Table S8.

#### 2.3.8 Synthesis of **CNO-PAS** Hybrid

CNO-SC(S)OEt (8.0 mg) was suspended in anhydrous THF (4.0 mL) and sonicated for 30 min. Then AIBN (0.03 mmol, 4.9 mg) and 4-acetoxystyrene (6.54 mmol, 1.0 mL) were added, and the reaction mixture was stirred for 24 h at 70 °C under Ar. The resulting suspension was then cooled and poured into cold MeOH, followed by filtration and drying *in vacuo* of the precipitate, affording 520.0 mg of the product as a black powder.

#### 2.3.9 Preparation of **CNO-PVPh** Hybrid

The CNO-PAS hybrid (472.0 mg) was suspended in MeOH (2.0 mL) and sonicated for 30 min. Then a 0.5 M solution of KOH in MeOH (2.0 mL) was added, and the reaction mixture was stirred for 20 h at 80 °C. Next, the reaction mixture was cooled, and the product was precipitated using 1 M HCl, followed by filtration, rinsing with distilled H_2_O, and oven-drying at 80 °C. The resulting product (400.0 mg) was a black powder.

#### 2.3.10 Polycondensation of **CNO-PVPh** with HCHO and F-127 (**CP-7**)

CNO-PVPh (385.0 mg) was suspended in a solution of Pluronic F-127 (385.0 mg) in 10% aqueous NaOH (4.0 mL) and EtOH (2.0 mL). The suspension was sonicated for 15 min, and it was stirred for 1 h at 90 °C while HCHO (4.0 mL) was added in portions. After this time, a precipitate was observed, and the reaction mixture was left at 90 °C without stirring for 19 h. The reaction mixture was then cooled, and the precipitate was filtered, washed with water, and dried in an oven at 100 °C overnight, affording 800.0 mg of the product as a green powder.

## 3 Results and Discussion

### 3.1 Synthesis of Star Polymers and Their Structural Characteristics

**6-***star-***(PMA**_*x*_**-***b***-PAS**_**200**_**)** (*x* = 25, 50, 100, 150) dendrimers, later transformed into **6-***star-***(PMA**_*x*_**-***b***-PVPh)**_**200**_, were synthesized by RAFT polymerization. The polymer-directed method of porous carbon material synthesis was performed, in which the sacrificial, pore-forming part was a block of PMA, undergoing thermal degradation at temperatures of approximately 300 °C.^27^ The carbonaceous skeleton was formed from poly(4-vinylphenol), which was cross-linked upon reaction with HCHO under environmentally friendly conditions in water. Dendrimers with different lengths of PMA chains were used to observe the effect of this feature on the pore size distribution in the synthesized carbonaceous materials.

**Scheme 1:**
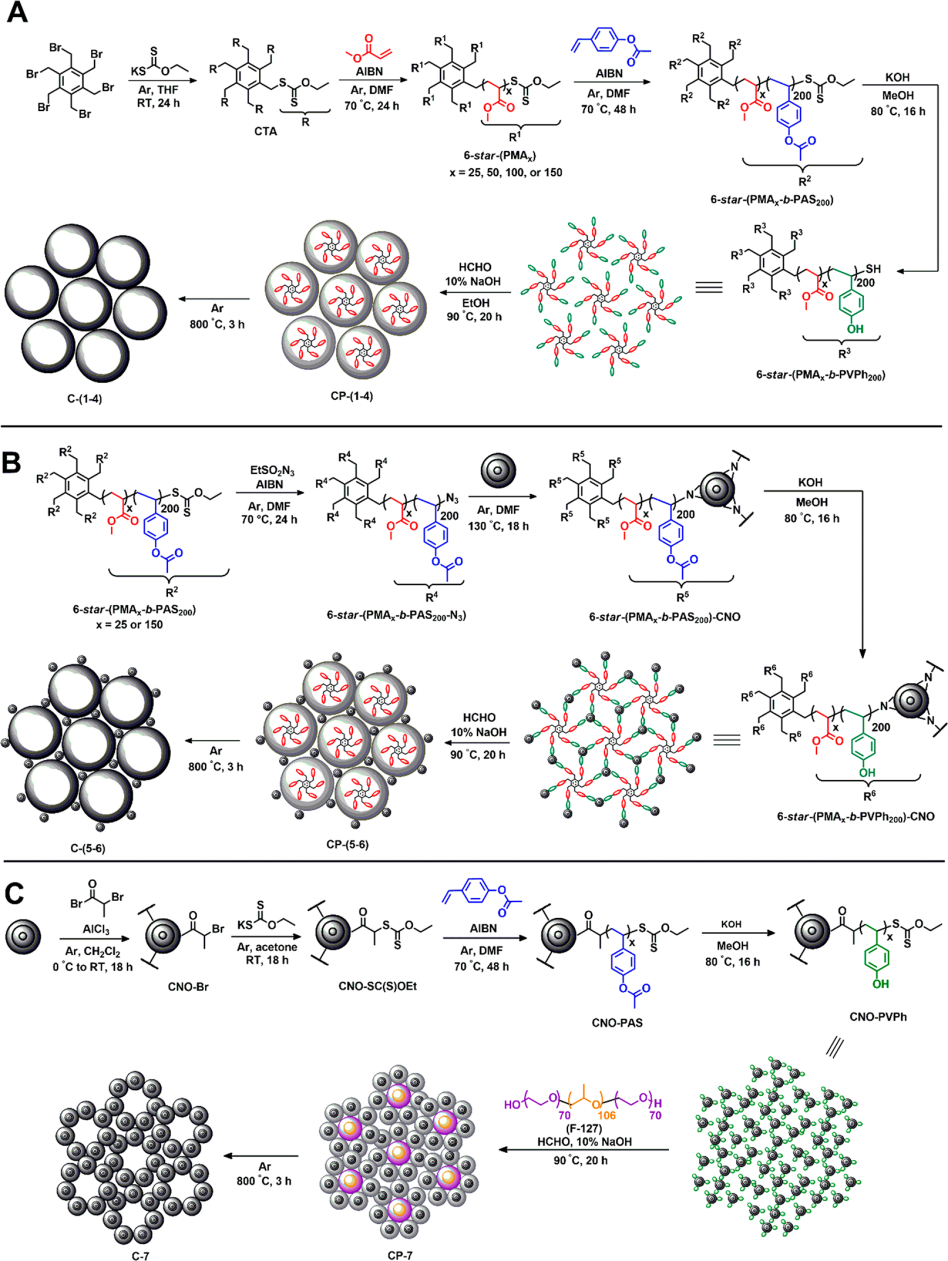
Representation of the Stepwise Synthesis of (A) PVPh-Derived Polymers and Carbon Materials from CTA, (B) CNO Modified with PVPh-Derived Polymers and Carbon Materials, and (C) CNO-Derived Chain Transfer Agent, Polymerization, and Carbon Material

The synthesis path was started from the preparation of a block star copolymer with a strictly defined structure employing RAFT polymerization (Scheme 1A). First, hexakis(*S*-methyl-*O*-ethyl dithiocarbonate)benzene was synthesized (see ^1^H and ^13^C NMR spectra in Figures S1 and S2) and used as a CTA. Next, the polymeric core was synthesized using the CTA and methyl acrylate with AIBN as an initiator of radical polymerization in DMF at 70 °C for 24 h (Scheme 1A and Table S1). The four resulting star polymers, **6-***star-***(PMA)**_*x*_, with different average arm lengths, theoretically equal to 25, 50, 100, and 150 monomer units per arm, were synthesized. The structure, molecular weight, and dispersity of the obtained polymers were determined by ^1^H NMR and SEC analyses (Table S9 and Figure S17). The average dendrimer arm length was calculated using ^1^H NMR spectra (Figures S3–S6) by the integration of the characteristic methylene −CH_2_– signal of the dithiocarbonate group (4.63 ppm) and the signal corresponding to the methyl −CH_3_ group of the PMA repeating unit (3.58 ppm). This value was used to calculate the molecular weights of the polymers. Furthermore, SEC analyses indicate the presence of star polymers with molecular weights increasing in the **6-***star-***(PMA)**_**25**_−**6-***star-***(PMA)**_**150**_ series and with a moderate dispersity index (*Đ*) with values of between 1.62 and 1.73.

The well-defined **6-***star-***(PMA)** polymers were subjected to RAFT polymerization with 4-acetoxystyrene and AIBN (Scheme 1A and Table S2). As a result, four different star polymers, **6-***star-***(PMA-***b***-PAS)**, were obtained and characterized by ^1^H NMR and SEC (Table S9 and Figure S17). We think that the integration of the characteristic −CH_2_– signal of the dithiocarbonate group at 4.63 ppm in the ^1^H NMR spectra (Figures S7–S10) is not reliable to be used for arm lengths and the molecular weight calculations for these long polymeric chains. Thus, only the PMA weight percent (wt % PMA) was calculated by integrating the signal at 3.58 ppm corresponding to the −CH_3_ group of poly(methyl acrylate) together with the multiplet in the range of 6.30–7.00 ppm corresponding to the aromatic protons of 4-acetoxystyrene groups. As planned, the mass fraction of sacrificial PMA blocks increases in the series (Table S9).

Due to the formation of a phenoxy radical, the phenol group is not compatible with radical polymerization; therefore, poly(4-acetoxystyrene) is commonly synthesized as a precursor of poly(4-vinylphenol).^28^ Therefore, at this stage of the synthesis, the 4-acetoxy groups of **6-***star***-(PMA-***b***-PAS)** polymers were deacetylated with a KOH solution in MeOH under the optimized conditions (Table S3), quantitatively affording four different 6-*star*-[poly(methyl acrylate)-*b*-poly(4-vinylphenol)], **6-***star***-(PMA-***b***-PVPh)**, polymers. Successful deprotection was confirmed by ^1^H NMR (Figures S11–S14), where signals from the acetyl group at 2.20 ppm were not observed and a signal from the hydroxy group at 8.95 ppm appeared. Interestingly, hydrolysis of the ester groups of the PMA blocks did not occur. Similarly to the case of **6-***star***-(PMA-***b***-PAS)** polymers, the evaluation of the PVPh block length of the **6-***star***-(PMA-***b***-PVPh)** was not estimated using ^1^H NMR spectra. Only the mass fraction of PMA was calculated by integrating the signal at 3.58 ppm (from the −CH_3_ groups of poly(methyl acrylate)) together with the multiplet in the range of 6.30–7.00 ppm (aromatic protons of 4-acetoxystyrene groups), increasing in the series (11, 18, 29, and 41 wt %, respectively) (Table S3).

The acrylic and styrenic polymers used in this work undergo decomposition in the temperature range between 300 and 550 °C.^29,30^ However, phenol–formaldehyde resins have a more temperature resistant carbon skeleton, and when they are pyrolyzed, they provide carbonaceous materials. Thus, the next step was the formation of a temperature-resistant structure using the synthesized star polymers. The polycondensation reaction of the phenolic groups with HCHO in basic media was used,^30^ inspired by the classical phenol–formaldehyde resin formation process.^31^**6-***star***-(PMA-***b***-PVPh)** polymers were subjected to polycondensation to afford four cross-linked star copolymers as carbon precursors, **CP-(1-4)** (Table S4). All precursors were pyrolyzed under an Ar flow for 3 h at 800 °C, giving the carbonized samples **C-(1-4)** (Scheme 1A).

The synthesis progress was monitored by FTIR spectroscopy of the products, which was described on the basis of the **C-4** material preparation. CTA has characteristic signals at 1217 and 1040 cm^–1^ corresponding to the stretching vibrations of the C–S group (Figure S18a).^32^ Due to the polymerization and incorporation into the CTA of many monomer molecules, these signals are obscured in the FTIR spectra. The FTIR spectrum of the **6-***star***-(PMA**_**150**_**)** polymer is dominated by the signal from the stretching vibrations of the C=O bond of the ester group at 1726 cm^–1^, a well-marked absorption band from 1120 to 1260 cm^–1^, which may be assigned to C–O–C bond stretching vibrations, and a band at 2953 cm^–1^ attributed mainly to CH sp^3^ vibrations (Figure S18b).^33^ The FTIR spectra of **6-***star***-(PMA**_**150**_**-***b***-PAS**_**200**_**)** show characteristic strong absorptions at 1732 cm^–1^ due to C=O stretching vibrations, bands at 1161 and 1187 cm^–1^ corresponding to strong Ph–O stretching absorptions, and a new band at 1504 cm^–1^ attributed to aromatic ring vibrations. Furthermore, the band at approximately 2950 cm^–1^ broadens due to numerous signals from new methyl groups (Figure S18c). The **6-***star***-(PMA**_**150**_**-***b***-PVPh**_**200**_**)** polymer, as expected, possesses a strong broad absorption band for −OH centered at 3368 cm^–1^. The signal from the C=O group present at 1715 cm^–1^ is shifted toward lower wavelengths and broadened due to the presence of nonbonded and hydrogen-bonded groups. Furthermore, in comparison to the intensity of the band at ca. 1500 cm^–1^ corresponding to the aromatic rings, which should be unchanged due to deacetylation, the intensities of the absorption attributed to the presence of the C=O groups and the C–O–C absorption at ca. 1200 cm^–1^ were decreased (Figure S18d).^33^ In the spectrum of **CP-4**, two broadened bands are visible, which may not be easily assigned to a specific group (Figure S18e). However, we can assume that the bands in the range between 1515 and 1700 cm^–1^ correspond to the vibrations of aromatic rings, −CH_2_–, and C=O. In turn, the band between 1240 and 1380 cm^–1^ may be attributed to the absorption of the C–O–C group in the polymeric skeleton. The peak at 1060 cm^–1^ may be assigned to stretching of the alkyl-phenyl ether. Furthermore, phenoxy group absorption was not visible in our FTIR study, probably due to the polycondensation reaction being performed under basic conditions and the formation of sodium phenoxide from phenol groups (Figure S18e). For the **C-4** sample, a significant decrease or disappearance of the absorption bands was observed, which is characteristic of a relatively pure carbon structure. However, one might assume that broad peaks at 1473 and 1655 cm^–1^ correspond to C=C aromatic vibrations, while the peak at 615 cm^–1^ represents ring bending vibrations (Figure S18f).^34^

### 3.2 Synthesis of the Covalent CNO-star Polymer Hybrids

The formation of the aziridine ring by the reaction of azides with carbon materials (e.g., fullerenes, carbon nanotubes) is a well-known method for the formation of covalent carbon–organic hybrids.^24^ To prepare CNO-star polymer hybrid materials (Scheme 1B), the previously prepared **6-***star***-(PMA-***b***-PAS)** polymers were used. The terminal functional group at the end of the star polymer arms, dithiocarbonate, was transformed into an azide with freshly prepared EtSO_2_N_3_ (Table S5).^35^ Reactions of radical azidation of xanthates were carried out on two selected **6-***star-***(PMA**_*x*_**-***b***-PAS**_**200**_**)** polymers with the shortest and the longest PMA chains in the structure (*x* = 25, 150). The successful synthesis of **6-***star***-(PMA**_**25**_**-***b***-PAS**_**200**_**-N**_**3**_**)** and **6-***star***-(PMA**_**150**_**-***b***-PAS**_**200**_**-N**_**3**_**)** was confirmed by ^1^H NMR (Figures S15 and S16) by monitoring the disappearance of the characteristic signals from the dithiocarbonate group, −CH_2_–, at 4.65 ppm. An analysis of the intermediate products leading to the target **C-(5-6)** was performed by FTIR spectroscopy and described using the example of the materials leading to **C-6** (Figure S19). The characteristic band from the vibrations of the azide group at 2130 cm^–1^ cannot be observed in the FTIR spectrum of **6-***star-***(PMA**_**150**_**-***b***-PAS**_**200**_**-N**_**3**_**)** because the mass fraction of −N_3_ groups in the polymer is very low, less than 5 wt % (Figure S19a).^36^

The polymers with azide groups, **6-***star-***(PMA-***b***-PAS-N**_**3**_**)**, were subjected to coupling with pristine CNOs using the optimized conditions (Table S6).^37^ The hybrids **6-***star-***(PMA**_**25**_**-***b***-PAS**_**200**_**)-CNO** and **6-***star-***(PMA**_**150**_**-***b***-PAS**_**200**_**)-CNO** were obtained as black powders insoluble in organic solvents. Their successful functionalization was confirmed by FTIR spectroscopy on the basis of the presence of characteristic functional groups of the polymeric stars linked to the CNOs (Figure S19b). The band at 1749 cm^–1^ was attributed to C=O vibrations, the band at 1504 cm^–1^ was related to the presence of aromatic rings, and the vibrations at 1170 cm^–1^ were assigned to C–O–C moieties.^38^

The next step started from deacetylation of the PAS chains of **6-***star-***(PMA-***b***-PAS)-CNO** hybrids, affording **6-***star-***(PMA**_**25**_**-***b***-PVPh**_**200**_**)-CNO** and **6-***star-***(PMA**_**150**_**-***b***-PVPh**_**200**_**)-CNO** (Table S7). The FTIR analysis confirmed the progress of this reaction by the appearance of vibrations related to the −OH group at 3362 cm^–1^, as well as a decrease in the intensity of the vibrations at 1714 cm^–1^ (C=O) and 1190 cm^–1^ (C–O–C) in relation to the band at 1510 cm^–1^, which corresponds to the C=C vibrations of aromatic rings (Figure S19c).^33^ A polycondensation step was then conducted (Table S8), resulting in carbon precursors **CP-5** and **CP-6**, characterized by absorption bands at 3673 cm^–1^ (−OH), 1828 cm^–1^ (bending C–H of aromatic rings), and 1516 cm^–1^ (C=C in the aromatic ring) (Figure S19d). Finally, pyrolysis at 800 °C was performed, which led to the transformation of organic materials into carbonaceous materials (**C-5** and **C-6**). For **CP-6**, two broad and intense bands at 1463 and 618 cm^–1^ were observed, which likely correspond to C=C aromatic vibrations or −CH_2_– methylene bridges and ring bends, respectively (Figure S19e).^34^

### 3.3 Polymers Derived from CNO-Based Chain Transfer Agents

A synthesis with an inverted sequence of the procedure was also performed (Scheme 1C), inspired by the “grafting from” strategy. In this approach, a chain transfer agent was synthesized by attaching dithiocarbonate groups to the CNOs and used to polymerize PAS. After PAS chain deacetylation, polymers were cross-linked with HCHO in the presence of micelles from the nonionic surfactant Pluronic F-127 used as a pore-forming agent and pyrolyzed.

A Friedel–Crafts acylation of pristine CNOs using 2-bromopropionyl bromide and AlCl_3_ as a catalyst in CH_2_Cl_2_ was used, affording **CNO-Br**.^39^ Excess acylating agent was used to ensure a selective reaction of only the more reactive acyl bromide group. The acylated CNOs were then subjected to a nucleophilic substitution reaction with potassium dithiocarbonate salt, and the CNO-derived chain transfer agent **CNO-SC(S)OEt** was obtained. Pristine CNO, **CNO-Br**, and **CNO-SC(S)OEt** were sonicated in Et_2_O or toluene for 1 h. The differences in dispersity can be noted depending on the structure of the CNO-derived materials and solvents used (Figure S20). A stable dispersion of **CNO-SC(S)OEt** in toluene and diethyl ether is indirect evidence of the successful functionalization of CNs. The **CNO-Br** vibration band at 1651 cm^–1^ assigned to C=O can be noted (Figure S21a). Moreover, the broad signals at 1631 and 1117 cm^–1^ and at 999 cm^–1^ may be attributed to C=O and dithiocarbonate groups for **CNO-SC(S)OEt**,^32^ respectively (Figure S21b).

A new chain transfer agent was used to polymerize 4-acetoxystyrene on its surface. The presence of PAS chains linked to CNOs (**CNO-PAS**) was confirmed by characteristic bands at 2925, 1766, 1504, and 1217 cm^–1^, which can be attributed to −CH, C=O, aromatic C=C, and Ph–O bonds of the PAS chains (Figure S21c), respectively.^38^ The next step of the synthesis was based on the deacetylation of PAS chains under basic conditions. A broad band at 3433 cm^–1^, corresponding to the vibrations of the −OH group, is observed (**CNO-PVPh**; Figure S21d), and simultaneously, the intensity of the band corresponding to the C=O group vibrations decreases significantly. Finally, CNOs grafted with PVPh chains were subjected to polycondensation with HCHO in the presence of a nonionic copolymer surfactant, Pluronic F-127, under basic conditions.^40^ F-127 creates micelles consisting of PPO blocks, which constitute a hydrophobic interior, and PEO blocks building a hydrophilic outer layer (Scheme 1C). Hydrogen bonds are formed between the −OH groups of the resin made of PVPh and HCHO and the ethylene oxide (EO) units repeating in the F-127 template. The self-organization of micelles and the **CNO-PVPh** moieties, the subsequent polycondensation of the phenol–formaldehyde type, and further carbonization resulted in the formation of the organized porous carbon skeleton. The resin, **CP-7**, has a characteristic signal at 2870 cm^–1^ attributed to C–H stretching vibrations and a band in the region from 1617 to 1482 cm^–1^, which is assigned mainly to the C=C aromatic bonds, as well as a band at 1096 cm^–1^ related to strong Ph–O vibrations and the strong C–O band of F-127 (Figure S21e).^41^ The last stage of the carbon material preparation was based on the pyrolytic transformation of the organic material into a carbon matrix (3 h, 800 °C), giving carbon material **C-7**, possessing one band assigned to C=C aromatic bonds and −CH_2_– moieties (Figure S21f).^34^

### 3.4 Structural and Thermal Characteristics of the CNO-star Polymer Derivatives

Pyrolysis at 800 °C under a constant Ar atmosphere leads to the transformation of the organic polymer chain into the carbon skeletons **C-(1-7)**. The details for the preparation of these samples are given in Table 1. The weight percentage of samples synthesized by the polycondensation reaction (**CP-(1-7)**) remaining after pyrolysis, **C-(1–7)**, was calculated (wt % pyrolysis). The mass fraction of CNOs in hybrid materials **C-(5-7)** (wt % CNO) was estimated by assuming that the mass of CNOs in the material undergoing thermal annealing does not change during the subsequent stages of the synthesis. The CNO content for all samples oscillates at around 5 wt %.

**Table 1:** Shortcut in the Preparation of PVPh-Derived Carbonaceous Materials^a^

synthesis		pyrolysis .		
polymer or hybrid	polycondensation product	pyrolysis product	wt % pyrolysis^b^	wt % CNO^c^
**6-***star***-(PMA**_**25**_**-***b***-PVPh**_**200**_**)**	**CP-1**	**C-1**	37.8	
**6-***star***-(PMA**_**50**_**-***b***-PVPh**_**200**_**)**	**CP-2**	**C-2**	39.4	
**6-***star***-(PMA**_**100**_**-***b***-PVPh**_**200**_**)**	**CP-3**	**C-3**	28.8	
**6-***star***-(PMA**_**150**_**-***b***-PVPh**_**200**_**)**	**CP-4**	**C-4**	22.6	
**6-***star***-(PMA**_**25**_**-***b***-PVPh**_**200**_**)-CNO**	**CP-5**	**C-5**	20.0	4.0
**6-***star***-(PMA**_**150**_**-***b***-PVPh**_**200**_**)-CNO**	**CP-6**	**C-6**	15.0	5.7
**CNO-PVPh (+ F-127)**	**CP-7**	**C-7**	17.6	5.0

a The lines of the table represent the corresponding products and should be interpreted as follows: the “polymer or hybrid” was the polycondensation substrate to give a “polycondensation product”, the pyrolysis of which gave the “pyrolysis product”.

b wt % pyrolysis = (*m*_C_:*m*_CP_) × 100%, where *m*_C_ is the mass of the sample obtained by pyrolysis and *m*_CP_ is the mass of the sample obtained by polycondensation.

c wt % CNO = (*m*_CNO_:*m*_C_) × 100%, where *m*_CNO_ is the mass of CNO subjected to the synthesis and *m*_C_ is the mass of carbon material.

Raman spectroscopy was used to analyze the selected polymeric, hybrid, and carbon materials. The low intensities at 842, 1174, 1197, and 1612 cm^–1^ may be assigned to the C–O–C,^42^ bending CH, stretching OH, and C=C vibrations of **6-***star***-(PMA**_**150**_**-***b***-PVPh**_**200**_), respectively (Figure S22A, spectrum a). The same polymer with the attached CNOs, **6-star-(PMA**_**150**_**-***b***-PVPh**_**200**_**)-CNO**, has an additional peak at 979 cm^–1^ related to a CH bend and three distinct peaks at 1308, 1612, and 2616 cm^–1^ (Figure S22A, spectrum b, and Table S10).^43^

The latter peaks are characteristic of the CN-disorder-induced D band, graphitic peak G, and 2D band, which is an overtone of the D band.^44^ Comparing the D to G intensity ratio (*I*_D_/*I*_G_) of the CNOs and the CNO–polymer hybrids provides information about the covalent functionalization of the CNOs with azide groups. The *I*_D_/*I*_G_ value of the pristine CNOs was equal to 2.42 (Figure S22B, spectrum e, and Table S10). After functionalization of the pristine CNOs, the number of aromatic bonds decreases, which results in a decrease in the *I*_D_/*I*_G_ ratio from 2.42 to 1.83.^45^ Both the polymer and CNO–polymer hybrids after pyrolysis, **C-4** and **C-6** (Figure S22A, spectra c and d), possess wide overlapping bands at 1310 and 1337 cm^–1^, respectively, which may be assigned to C atoms with sp^3^ hybridization, and bands at 1583 and 1590 cm^–1^, respectively, attributed to vibrations of the sp^2^-hybridized carbon atoms. These vibrations are characteristic of carbonaceous materials and are attributed to the transformation of organic polymers to the carbon skeleton.^46^ Due to the overlapping of these vibrations with the D and G bands of the CNOs, the vibrations of **C-6** cannot be well-defined.

The Raman spectra of CNO, **CNO-SC(S)OEt**, **CNO-PAS**, and **C-7** are shown in Figure S22B. An *I*_D_/*I*_G_ value equal to 2.27 for **CNO-SC(S)OEt** (Table S10) is lower than the *I*_D_/*I*_G_ value of the pristine CNOs (*I*_D_/*I*_G_ = 2.42). These features may indicate the successful covalent functionalization of the CNs with the 2-bromopropionyl substituent. Weak signals at 810 and 847 cm^–1^ (C–O and ring deformation), 1168 and 1203 cm^–1^ (C–O and bending C–H), 1446 cm^–1^ (symmetric C–C and bending C–H), and 1759 cm^–1^ (C=O) confirm the presence of poly(4-acetoxystyrene) in the **CNO-PAS** derivative (Figure S22B, spectrum g).^47^ The *I*_D_/*I*_G_ ratio is equal to 1.84. For **C-7** (Figure S22B, spectrum h), the wide and overlapping bands at 1301 and 1596 cm^–1^ attributed to C sp^3^ and sp^2^ vibrations, respectively, are present due to pyrolysis and the formation of carbon materials.^46^ The samples containing CNOs (**6-***star***-(PMA**_**150**_**-***b***-PVPh**_**200**_**)-CNO**, **CNO-SC(S)OEt**, and **CNO-PAS**) also exhibit broad bands at 2616, 2596, and 2598 cm^–1^, respectively (Table S10). These bands are assigned to overtone scattering (∼1299 cm^–1^ × 2 and ∼1596 cm^–1^ × 2) and combinational scattering (∼1299 cm^–1^ + ∼1596 cm^–1^).

XPS measurements were used to determine the elemental composition of the surfaces of **C-1**, **C-4**, and **C-(5-7)** (Table S11). The XPS measurements indicate that all tested samples contain C, O, and Na. For both samples, with and without CNOs (**C-1** with **C5** and **C-4** with **C-6**), the amount of C relative to O increases slightly with the chain length of the PMA. Sodium is derived from the salt, sodium phenoxide, into which some of the phenol groups of PVPh chains have been converted as a result of a polycondensation reaction in aqueous 10% NaOH. The presence of N in the **C-(5-7)** samples is related to the aziridine ring linking the star polymers with CNOs.

**Figure 1:**
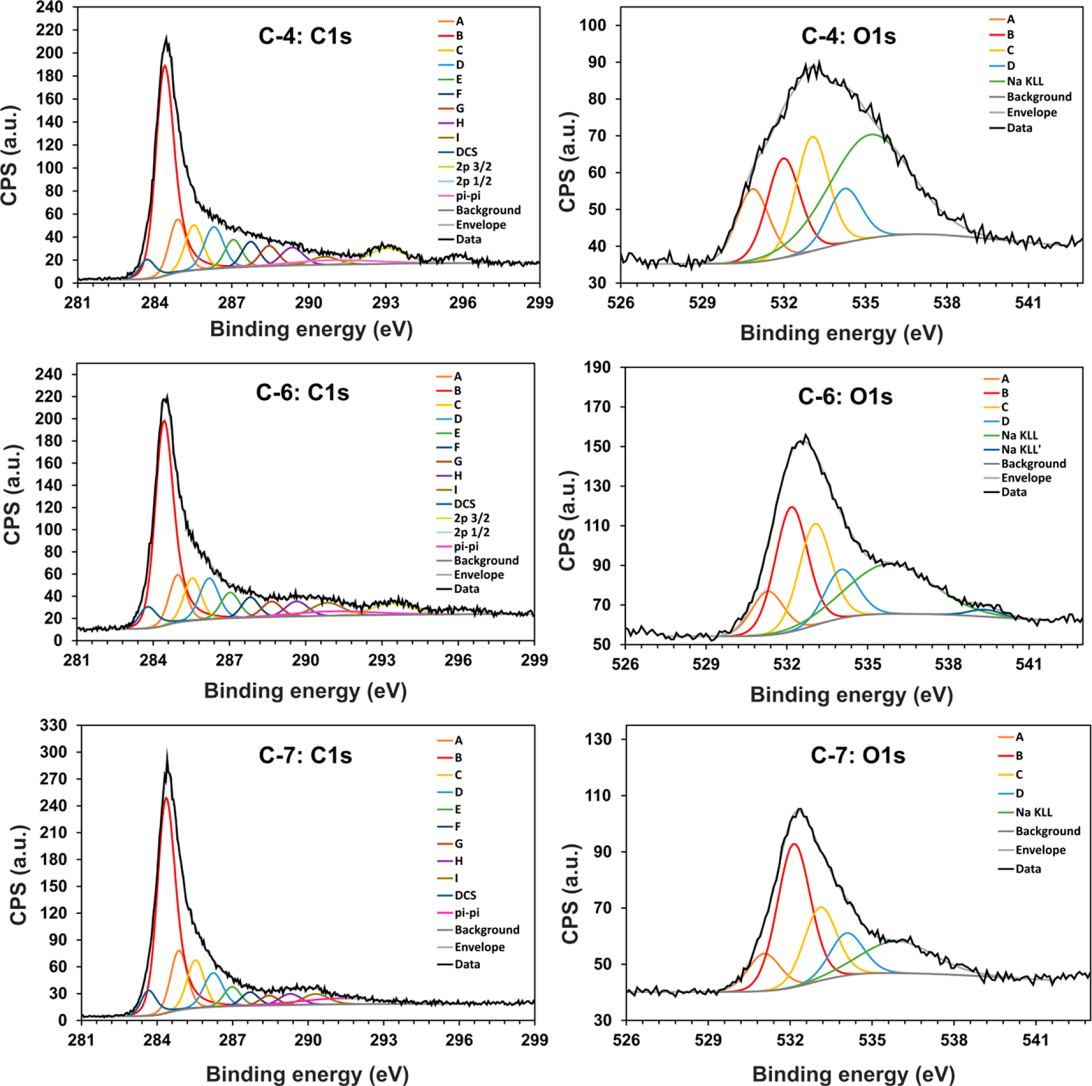
XPS spectra of the C 1s and O 1s spectral regions of the **C-4**, **C-6**, and **C-7** samples.

The distribution of heteroatom species was obtained from the deconvolution of the high-resolution spectral regions C 1s, O 1s, and N 1s and is presented in Figure 1 and Figure S23. The assignments and percentages of constituents are summarized in Table 2, and the details of curve fitting are provided in Tables S12–S14. The deconvolution of XPS C 1s spectra for all samples indicates a similar pattern of distribution of elements. The peak at ∼284.3 eV of sp^2^-hybridized C (C=C) is dominant, which is typical for aromatic rings (Table 2). The peaks at ∼284.9, ∼285.5, and ∼286.3 eV correspond to the C–H sp^3^ and C–C sp^3^ bonds of aliphatic C, as well as C–OH or C–N moieties, and constitute 8–12% of the surface concentration. The smallest amount of C (in the range between 2% and 8%) can be attributed to the ether (∼287.0 eV), carbonyl (∼287.7 eV), and carboxyl groups (∼288.4, ∼289.4 eV), a π–π* transition (∼290.3 eV, in aromatic conjugated systems), defects in the CNs, and vacancy-like defects in the graphitic lattice (∼283.6 eV). Most of these moieties are typical for methylene and methylene ether linkers and methyl, hydroxymethyl, aldehyde, ketone, quinone, and phenol groups characteristic of resorcinol–formaldehyde resin.^31^

**Table 2:** Distribution of Elements Obtained from the Deconvolution of the C 1s, O 1s, and N 1s Spectra by XPS

					concentration (atom %)				
region	peak	BE (eV)	assignment	ref	**C-1**	**C-4**	**C-5**	**C-6**	**C-7**
C 1s	A	284.9 ± 0.1	C–H sp^3^	(61)	10.1	10.2	9.6	9.4	11.9
	B	284.3 ± 0.1	C=C sp^2^	(61, 62)	52.9	46.9	40.5	46.9	50.6
	C	285.5 ± 0.1	C–C sp^3^	(61, 62)	8.5	9.0	9.1	8.6	9.9
	D	286.3 ± 0.1	C–OH, C–N (aziridine)	(48, 61, 62)	7.7	9.1	8.6	9.1	7.8
	E	287.0 ± 0.1	C–O–C	(61, 62)	4.0	5.4	6.5	5.5	3.9
	F	287.7 ± 0.1	C=O	(61, 62)	3.2	4.9	4.1	4.0	2.8
	G	288.4 ± 0.1	O=C–O–	(62, 63)	3.1	4.2	4.4	3.5	2.1
	H	289.4 ± 0.1	O=C–OH	(63)	2.9	4.3	8.3	3.7	2.8
	I	290.3 ± 0.1	π–π*	(38)	3.4	2.4	3.4	4.5	3.2
	DCS	283.6 ± 0.1	defects in carbon structure	(64)	4.3	3.7	5.5	4.8	5.0
O 1s	A	531.2 ± 0.1	C=O	(65)	26.1	21.2	37.8	13.6	12.4
	B	532.2 ± 0.1	C–OH/epoxy	(66, 67)	40.6	29.3	37.6	39.2	48.5
	C	533.2 ± 0.1	C–O–C	(66, 68)	20.1	33.3	17.8	31.6	24.6
	D	534.2 ± 0.1	Ph–OH, O=C–O–	(65, 68)	13.2	16.3	6.8	15.6	14.6
N 1s	A	398.3	pyridinic N	(69, 70)				18.4	
	B	399.8	aziridine	(48, 49)				51.5	
	C	401.3	quaternary N	(69, 70)				20.2	
	D	403.3	pyridinic N oxide	(69, 70)				9.9	

The deconvoluted XPS O 1s regions indicate the presence of mainly −OH groups or epoxides (∼532.2 eV) and confirm the formation of C=O (∼531.2 eV), ether (∼533.2 eV), and phenol/carboxyl species (∼534.2 eV), in line with the structure of phenol–resorcinol resin.^31^ An N 1s XPS analysis was only successfully performed for the **C-6** sample due to the excessively low amount of N in the other materials, **C-5** and **C-7** (Figure S23). The XPS spectrum for **C-6** can be resolved into four regions, which correspond mainly to the aziridine ring (399.8 eV). This signal indicates the formation of covalent linkages between CNOs and organic azides.^53^ Furthermore, peaks at 398.3, 401.3, and 403.3 eV were identified, which can be assigned to pyridinic, quaternary N, and pyridinic N oxides, respectively. These functional groups may result from the rearrangements of the aziridine ring at high temperatures and the incorporation of N into aromatic carbon structures.

**Figure 2:**
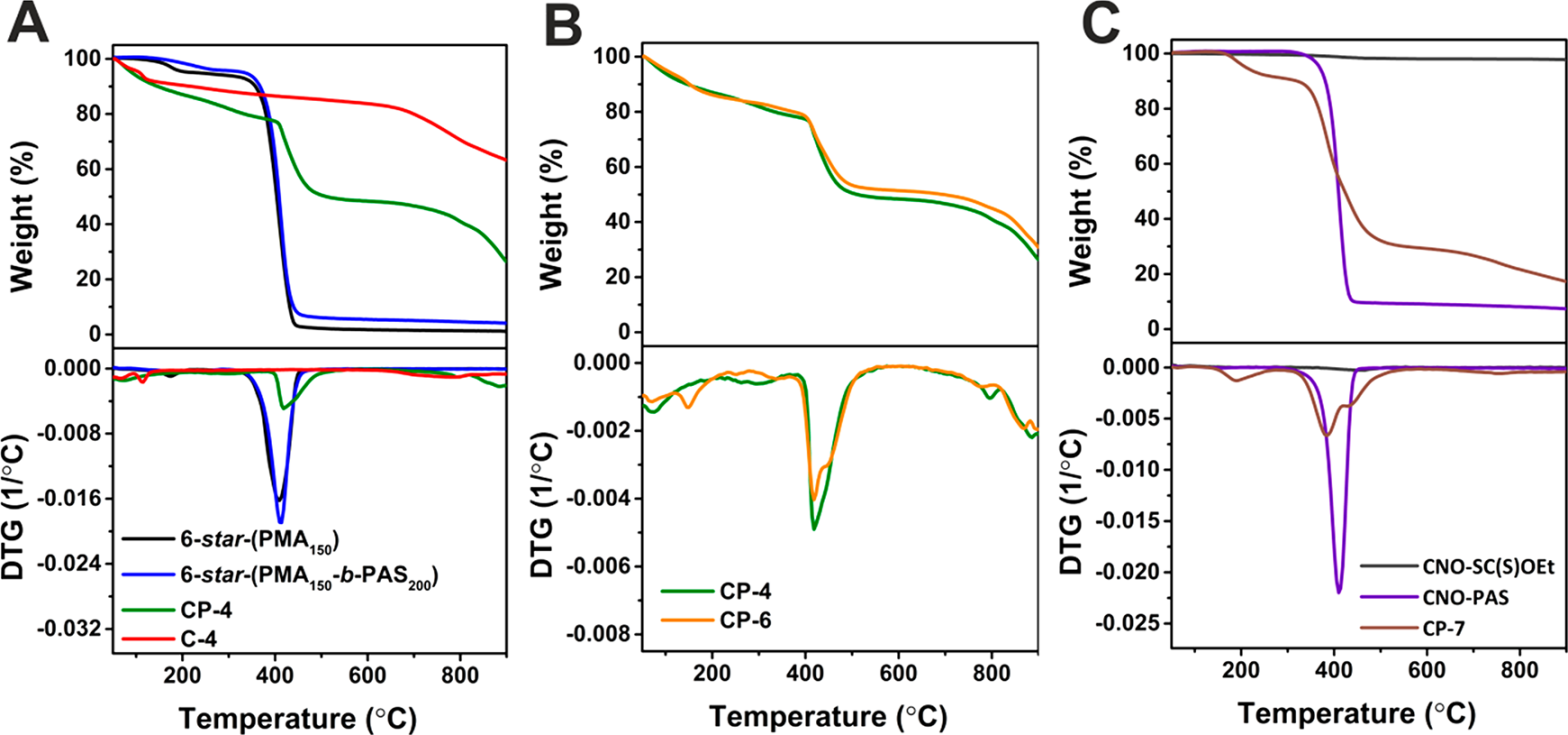
TG curves (top panel) and DTG curves (bottom panel) of (A) **6-***star***-(PMA**_**150**_**)**, **6-***star***-(PMA**_**150**_**-***b***-PAS**_**200**_**)**, **CP-4**, and **C-4**, (B) **CP-4** and **CP-6**, and (C) **CNO-SC(S)OEt**, **CNO-PAS**, and **CP-7**.

The thermal stability of the selected materials was studied using thermogravimetric analysis (TGA). The polymers **6-***star***-(PMA**_**150**_**)** and **6-***star***-(PMA**_**150**_**-***b***-PAS**_**200**_**)** decompose almost totally, showing one significant weight loss between 350 and 450 °C with the maximum degradation rate being at 410 °C (Figure 2A). The cross-linked resin (**CP-4**) and pyrolyzed product (**C-4**) show different decomposition profiles, degrading continuously over the entire temperature range applied. However, the TGA of **CP-4** shows two decomposition steps (in ranges 400–500 and 750–900 °C), whereas **C-4** shows only one distinguishable weight loss at higher temperatures between 750 and 900 °C. The first step of **CP-4** degradation is related to the depolymerization of PMA chains. On the other hand, the second step shows that our materials obtained at the pyrolysis temperature of 800 °C may undergo further changes at a temperature higher than 800 °C. The residues observed on the TG curves of the materials after the polycondensation reaction and pyrolysis confirm the formation of carbonaceous materials, which do not decompose under an inert atmosphere in the applied temperature range (Figure 2A and Table S15).

TG and DTG curves of **CP-4** and **CP-6** are presented in Figure 2B. As expected, the materials have similar degradation profiles and two decomposition steps with maximum degradation rates at approximately 420 and 880 °C (Figure 2B). The difference is the amount of residue observed at 900 °C; the amount of residue for **CP-4** is 26%, and the amount of residue for **CP-6** is 31%. A larger amount of residue in the case of **CP-6** is most likely related to the presence of CNOs that do not decompose up to 900 °C under an inert atmosphere. On the TG curve of dithiocarbonate-functionalized CNOs (**CNO-SC(S)OEt**), only one small weight loss (2%) at approximately 420–480 °C is observed (Figure 2C), which is indirect evidence of the functionalization of the CNO’s surface. In contrast, the **CNO-PAS** sample degrades almost quantitatively, showing one significant weight loss (93%) in the temperature range between 350 and 450 °C, which can be attributed to the depolymerization of PAS and the residue (7%) identified as CNOs. After the polycondensation reaction (**CP-7**), several decomposition steps with maximum degradation rates at 190, 380, 430, and 760 °C were observed. The first degradation step that differs from the other **CP** samples curves corresponds to the decomposition of Pluronic F-127.^50^ The changes in the thermal decomposition profiles and amounts of TG residues observed for samples after polymerization and polycondensation steps confirm CNO’s functionalization.

**Figure 3:**
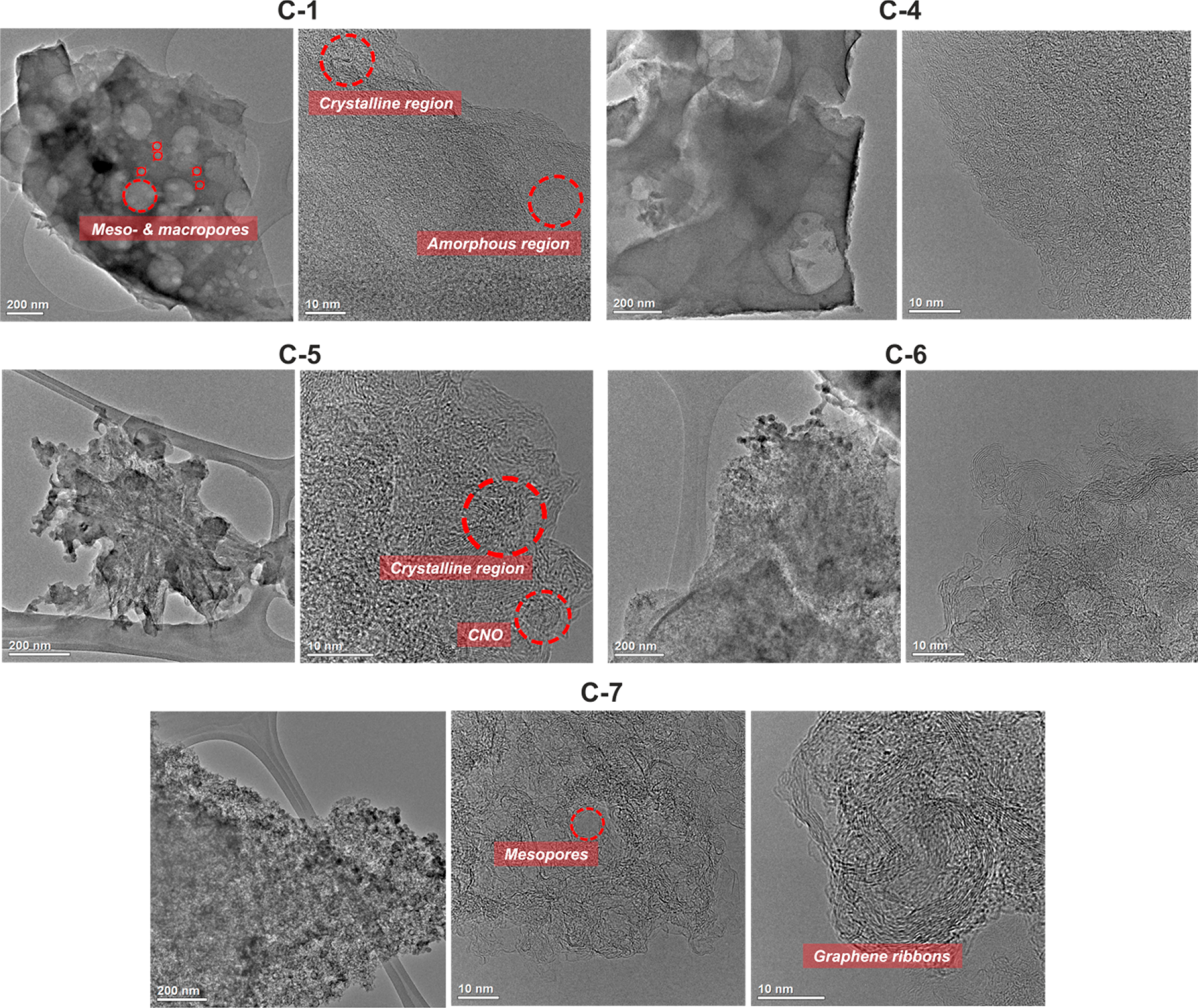
HRTEM images of the **C-1**, **C-4**, **C-5**, **C-6**, and **C-7** samples.

HRTEM studies were performed to analyze the carbonaceous materials (Figure 3). The samples derived from **6-***star***-(PMA**_*x*_**-***b***-PVPh**_**200**_**)** star copolymers (**C-1** and **C-4**) have uniformly distributed spherical cavities in the structure, which may be assigned to macropores (>50 nm), known for helping the access of the solution of electrolyte and the movement of ions under the external electrical potential,^51^ as well as spherical cavities with a size corresponding to mesopores (2–50 nm). Moreover, apart from amorphous phases, crystalline zones can also be observed, which are the reason for the higher structural stability, higher conductivity, and Coulombic efficiency of carbon materials.^52^ HRTEM studies of the samples containing CNOs (**C-5**, **C-6**, and **C-7**) clearly show the presence of multilayered fullerenes in the structures and a beneficial increase in the crystallinity of materials containing CNOs in comparison to pyrolyzed pristine polymers. In particular, sample **C-7** exhibits higher homogeneity and crystallinity in comparison to the other samples. The second feature is clearly illustrated by the presence of graphene ribbons,^53^ indicating that the use of the polymer–carbon hybride formation method, involving the grafting of polymers on the functionalized CNOs (**C-7**), has an advantage over the method of covalent bonding of presynthesized polymers and CNs (**C-5** and **C-6**). HRTEM of **C-7** at a higher magnification shows hollow spheres with a size of approximately 5–7 nm, which confirms the presence of a mesoporous material. Additional HRTEM images are given in Figures S24 and S25.

**Figure 4:**
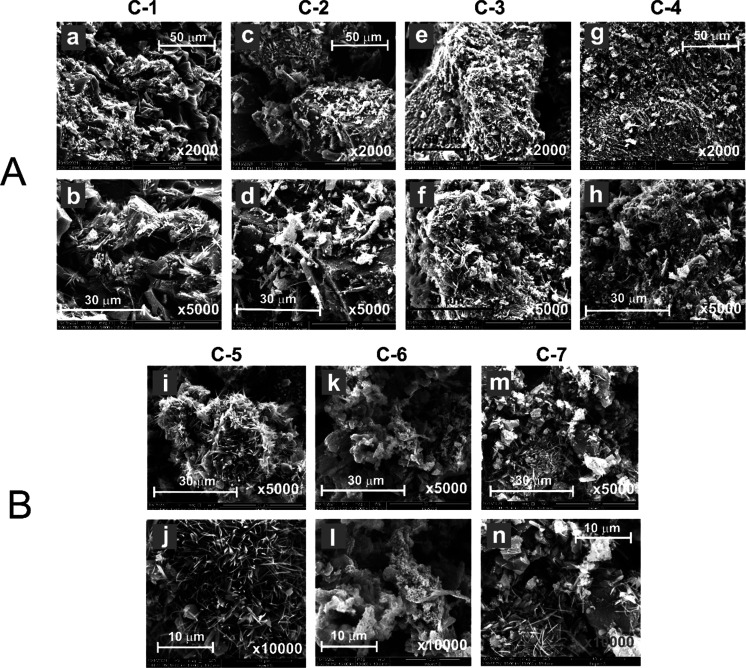
SEM images with different magnifications of (A) pyrolyzed star copolymers (a, b) **C-1**, (c, d) **C-2**, (e, f) **C-3**, and (g, h) **C-4** and (B) pyrolyzed CNO–polymer hybrids (i, j) **C-5**, (k, l) **C-6**, and (m, n) **C-7**.

The morphology of all the pyrolyzed star copolymers (from **C-1** to **C-4**) and the CNO-polymer hybrids (**C-5**, **C-6**, and **C-7**) prepared as films on a conducting surface was studied using SEM. The SEM images of pyrolyzed **6-***star***-(PMA**_*x*_**-***b***-PVPh**_**200**_**)** with different lengths of PMA chains (*x* = 25, 50, 100, 150) are presented in Figure 4A. The images obtained for **C-1** (Figure 4A-a,b) confirm the presence of two morphological forms in its structure: namely, needlelike structures and a spongelike network. An uneven distribution of both forms in the carbon materials resulted in their relatively low homogeneity. The characteristic features of **C-1** are numerous holes between the aggregates and sharp edges of the CNs. Increasing the number of monomer units in PMA chains used for the synthesis of **C-2** resulted in significant changes in the morphology of the related carbon material (Figure 4A-c,d) in comparison to **C-1**. Increasing the needlelike structures in the material tended to lead to their aggregation. Moreover, the number of pores in the spongelike structure also increased, which greatly increased the porosity and homogeneity of **C-2**. The SEM studies of **C-3** (Figure 4A-e,f) clearly show the dominance of filamentous organic forms, which probably completely cover the surface of the spongelike network. This material exhibited a more compact, aggregated, and porous structure in comparison to **C-1** and **C-2**. Additionally, SEM studies of **C-4** (Figure 4A-g,h) confirmed that it is the most porous of all materials. In summary, we can state that the PMA chain length in the structure of the star copolymers after pyrolysis is an extremely important factor that controls the morphology of these materials. The longer the PMA blocks in the copolymer, the greater the porosity of the synthesized materials.

The structural differences were even more pronounced in the case of carbon materials derived from hybrids containing selected **6-***star***-(PMA**_*x*_**-***b***-PVPh**_**200**_**)** and CNOs (**C-5** and **C-6**) and CNOs grafted with PVPh (**C-7**). **C-5** showed the dominance of characteristic aggregates of thin needlelike structures growing in different directions (Figure 4B-i,j). Similar forms were observed for the **C-1** copolymer, but the presence of CNOs in the **C-5** sample increased the number of needles and improved the homogeneity of the hybrid material, proving the beneficial influence of the CNOs on the organization of polymer chains. Moreover, similar conclusions can be drawn by analyzing the morphological features of the material derived from the star copolymer with the longest average PMA chains (**C-4**) and analogous material with the CNOs (**C-6**). The flakelike 3D structure of **C-6** (Figure 4B-k,l) seems to be less porous, compact, and more homogeneous than the **C-4** structure. Interestingly, the pyrolyzed CNOs grafted with PVPh (**C-7**), obtained by a different synthetic approach (Scheme 1C), showed morphological features intermediate between those of the **C-5** and **C-6** structures. Briefly, for the **C-7** sample, flake-shaped aggregates similar to those in the **C-6** structures were observed (Figure 4B-m,n), on which needlelike structures, characteristic of the **C-5** sample, were located.

### 3.5 Textural Characteristics of the CNO-star Polymer Derivatives

**Figure 5:**
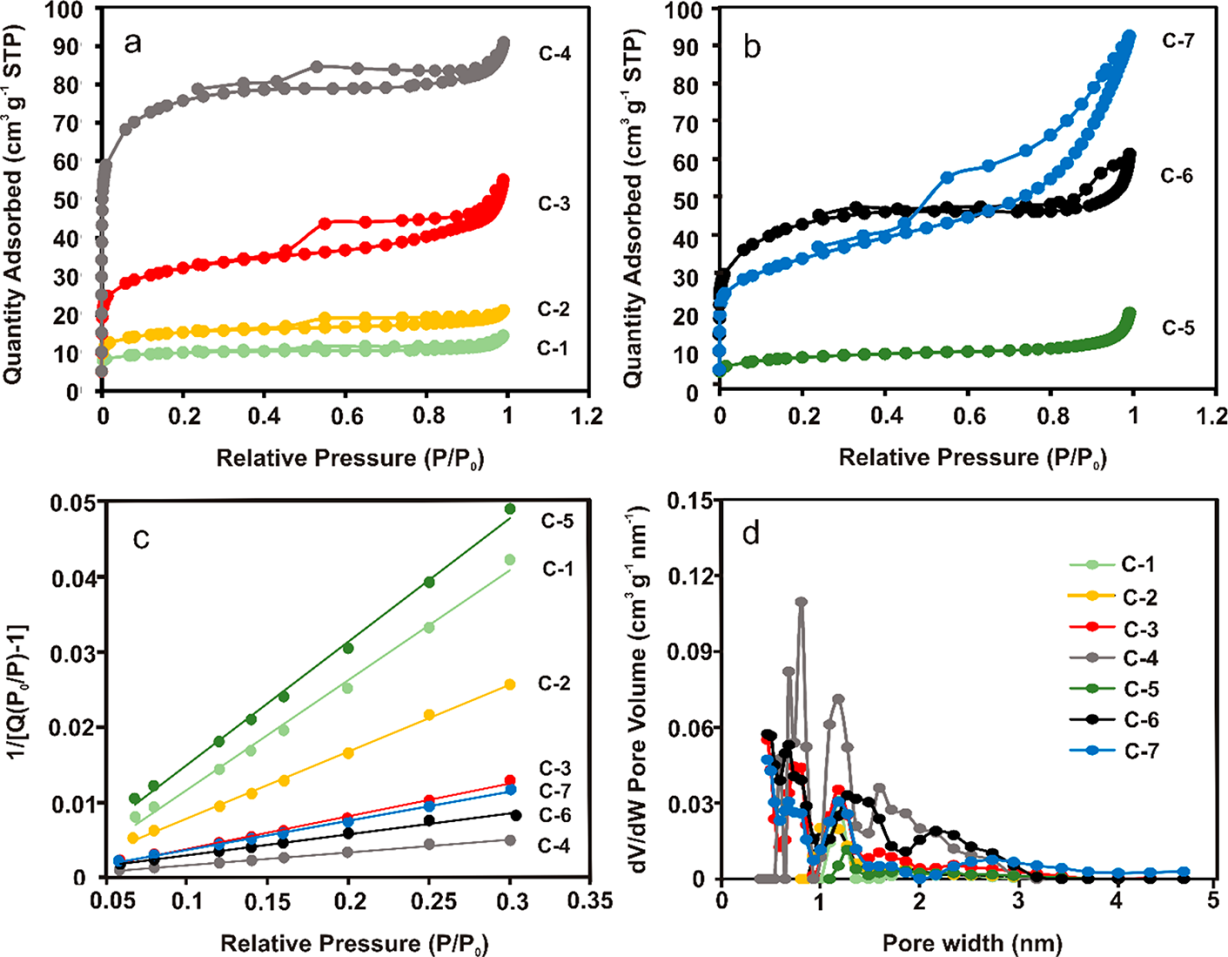
Nitrogen adsorption/desorption isotherms of pyrolyzed (a) **C-1**, **C-2**, **C-3**, and **C-4**, and (b) **C-5**, **C-6**, and **C-7**, (c) the BET curves, and (d) the pore volume distribution from the DFT model obtained for all materials.

The N_2_ adsorption/desorption isotherms of pyrolyzed **6-***star***-(PMA**_*x*_**-***b***-PVPh**_**200**_**)** with different average lengths of PMA chains are shown in Figure 5a. The course of the recorded curves was a combination of type I isotherms (sharp growth below 0.05 *P*/*P*_0_) and type IV isotherms (hysteresis loop in the *P*/*P*_0_ range of 0.45–0.9), indicating the coexistence of micro- and mesopores in the structure of the carbon materials.^54^ The hysteresis loop observed for all isotherms (Figure 5a), according to the IUPAC nomenclature, was classified as the H4 type, which indicated the presence of narrow mesopores in the **C-(1-4)** samples.^55^ A study of the porosity of the **C-(1-4)** samples clearly showed the significant influence of the PMA chain length on the amount of N_2_ adsorbed on the surface of the materials. An analysis of isotherms carried out in a narrow range of *P*/*P*_0_ (0.05–0.3) (Figure 5c), on the basis of the Brunauer–Emmett–Teller (BET) theory,^56^ allowed us to determine the specific surface area (*S*_BET_) values of the **C-(1-4)** carbon materials (Table 3). As expected, **C-4** showed the highest *S*_BET_ value (247 m^2^ g^–1^) due to the presence of the longest PMA chains (Table 3). These results are consistent with the SEM studies (Figure 4A), which clearly show that porosity in the series from **C-1** to **C-4** was increased. Moreover, a *t* plot analysis showed the advantage of the number of micropores over mesopores in all pyrolyzed copolymers (Table 3). An increase in the average pore diameter, with a simultaneous increase in the pore volume upon an increase in the length of the PMA chains in the **C-(1-4)** structures, was also observed. The change in the pore diameter most likely results from the differences in the geometry of the used dendrimers. Furthermore, the porosity of the pyrolyzed CNO–polymer hybrids (**C-5**, **C-6**, and **C-7**) significantly differed from the texture of the corresponding copolymers (**C-1** and **C-4**). First, the shape of the isotherms recorded for **C-5** and **C-6** hybrids with the H3 hysteresis loop in the *P*/*P*_0_ range from 0.8 to 1 indicated the mesoporous nature of the materials (Figure 5b).^57,58,61^ However, the N_2_ adsorption/desorption curve obtained for the **C-7** sample (Figure 5b) showed common features with isotherms of all pyrolyzed copolymers (hysteresis loop in a wide range of *P*/*P*_0_) and **C-(5-6)** (no limiting adsorption at maximum *P*/*P*_0_).

**Table 3:** Textural Parameters of the Synthesized Materials Calculated from N_2_ Adsorption/Desorption Studies

sample	*S*_BET_ (m^2^ g^–1^)	external surface area^a^ (m^2^ g^–1^)	micropore area^b^ (m^2^ g^–1^)	pore volume (cm^3^ g^–1^)	av pore diameter^j^ (nm)
**C-1**	31	11	20	0.021^c^	4
**C-2**	50	18	32	0.031^d^	5
**C-3**	102	54	47	0.080^e^	6
**C-4**	247	98	149	0.136^f^	6
**C-5**	27	23	4	0.027^g^	8
**C-6**	146	82	64	0.089^h^	7
**C-7**	113	84	32	0.139^I^	8

a Derived from a *t* plot.

b Calculated from a *t* plot.

c Single-point adsorption total pore volume of pores less than 1193.503 Å diameter at *P*/*P*_0_ = 0.983656109.

d Single-point adsorption total pore volume of pores less than 1249.539 Å diameter at *P*/*P*_0_ = 0.984403530.

e Single-point adsorption total pore volume of pores less than 1196.945 Å diameter at *P*/P_0_ = 0.983704080,

f Single-point adsorption total pore volume of pores less than 1297.256 Å diameter at *P*/*P*_0_ = 0.984988222,

g Single-point adsorption total pore volume of pores less than 1282.640 Å diameter at *P*/*P*_0_ = 0.984813825,

h Single-point adsorption total pore volume of pores less than 1303.034 Å diameter at *P*/*P*_0_ = 0.985056066,

I Single-point adsorption total pore volume of pores less than.1223.239 Å diameter at *P*/*P*_0_ = 0.984061418;

j Calculated from BJH adsorption average pore width (4 V/A).

Moreover, the *S*_BET_ values for pyrolyzed hybrids **C-(5-6)** (Figure 5c and Table 3) suggest that incorporation of the CNOs into the polymeric network reduced their *S*_BET_ in comparison to the corresponding pristine copolymers (*S*_BET_ of **C-5** is 27 m^2^ g^–1^ vs *S*_BET_ of **C-1** 31 m^2^ g^–1^; *S*_BET_ of **C-6** 146 m^2^ g^–1^ vs *S*_BET_ of **C-4** 247 m^2^ g^–1^). The *S*_BET_ value determined for **C-7** was 113 m^2^ g^–1^ (Table 3), which was between the values obtained for the remaining hybrids. The analysis of other textural parameters (Table 3), in turn, indicates an increase in the mesoporosity of all CNO–polymer hybrids and a decrease in the number of micropores. With the isotherm as a starting point, it is possible to correlate the necessary gas volume to fill all the cylindrical pores in the materials, according to the formula
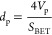
1where *d*_p_ is the average pore diameter (nm), *V*_p_ is the pore volume (cm^3^ g^–1^), and *S*_BET_ is the specific surface area (m^2^ g^–1^). The pore volume, *V*_p_, increased from 0.021 to 0.136 cm^3^ g^–1^ in the **C-(1-4)** series and from 0.027 to 0.139 cm^3^ g^–1^ in the **C-(5-7)** series (Table 3).

In addition, the pore-size distribution in the **C-(1-7)** samples, calculated both by the Barrett–Joyner–Halenda method (BJH) (Table 3) and by nonlocal density functional theory (NLDFT) (Figure 5d), indicates their wide range distribution and thus some degree of irregularity. Figure 5d presents the distribution of pores in a narrow range of widths for all materials. Analyses show an increase in the number of pores with diameters above 2 nm (mesopores) for samples containing CNOs, which confirms the growth in mesoporosity of the hybrid materials.

### 3.6 Electrochemical Performance of the Carbon Materials

The capacitance of carbon materials depends on the porosity of the films, the defects on the surface of the CNs, and their chemical heterogeneities. These parameters significantly influence the electrochemical behavior of the porous materials and contribute to the increase in the total capacitance of EDLCs. Solid films of the carbon materials were prepared using the drop-coating method. A drop of the dispersion in EtOH and CP containing the carbon materials was deposited on the GCE surfaces. The GCE electrode modified with a subsequent carbon material was then transferred to an organic solution with 0.1 M supporting electrolyte. Under these conditions, the films exhibited excellent electrochemical stability under cyclic voltammetry (CV).

**Figure 6:**
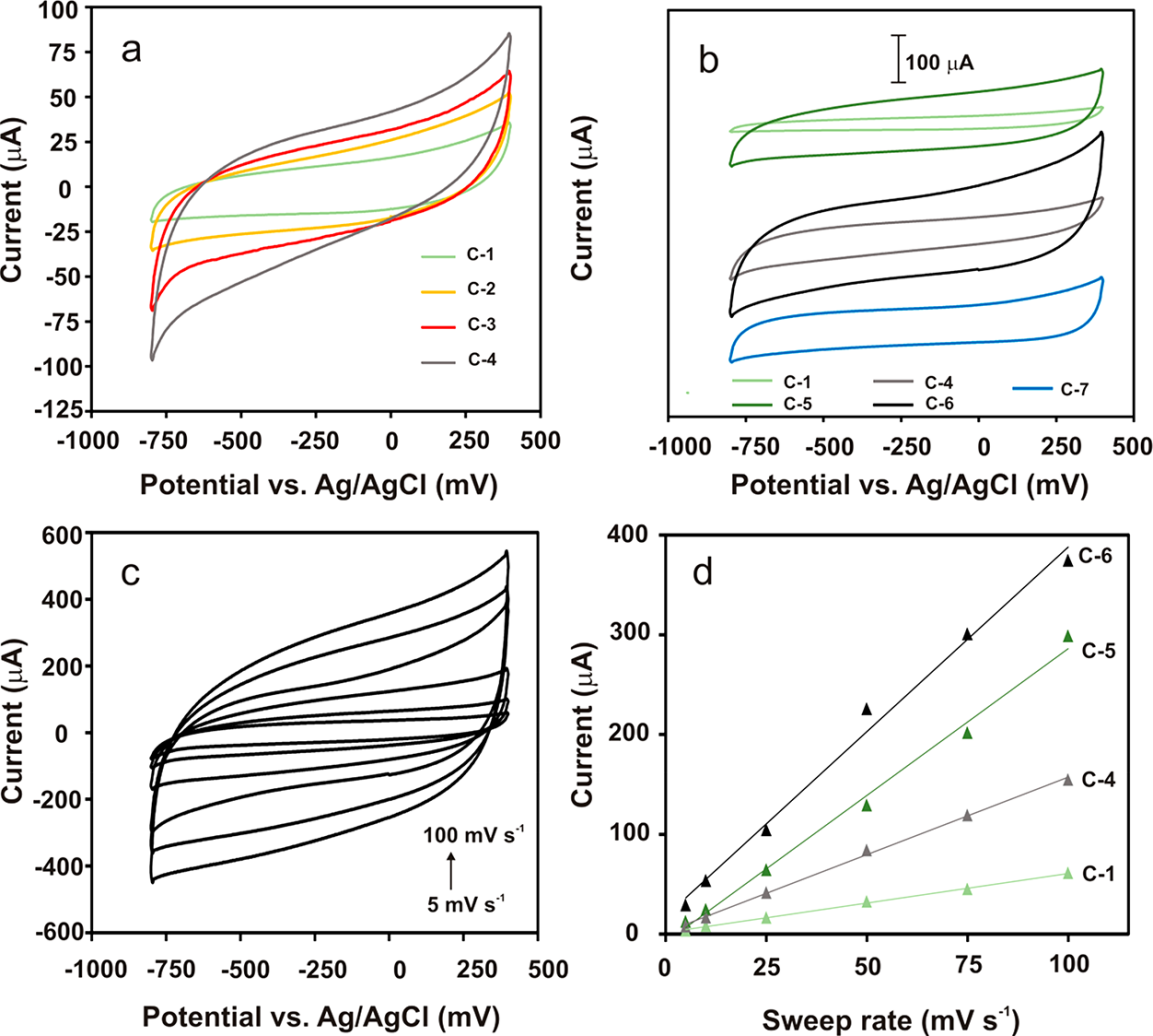
CVs of GCE modified with (a) **C-1**, **C-2**, **C-3**, and **C-4** and (b) **C-5**, **C-6**, and **C-7** carbon materials recorded in ACN containing 0.1 M TBAPF_6_ at a scan rate of 20 mV s^–1^. (c) CVs of a GCE modified with **C-6** recorded in ACN containing 0.1 M TBAPF_6_ at different scan rates (5, 10, 25, 50, 75, and 100 mV s^–1^). (d) Dependence of *i*_c_ on *v* for **C-1**, **C-4**, **C-5**, and **C-6** at −200 mV vs Ag/AgCl.

The electrochemical properties of the GC electrode modified with the pyrolyzed copolymers, **C-(1-4)**, and the pyrolyzed CNO–polymer hybrids, **C-(5–7)**, were examined in an ACN solution with 0.1 M TBAPF_6_ as the supporting electrolyte using the CV method (Figure 6a,b). The CVs of all materials show rectangular cathodic and anodic profiles in the potential range between −800 and +400 mV vs Ag/AgCl, suggesting the capacitive character of all investigated films. However, it should be emphasized that in the case of electrodes modified with the **C-(1-4)** samples (Figure 6a), some deviations from the rectangular shape of the CVs are observed, probably due to the charge-transfer resistance of the films. The longer the PMA chains in the carbon materials and the higher the *S*_BET_ value with a higher content of micropores, the more pronounced the deviations observed from the ideal shape of the CVs were.

An increase in the length of the PMA chains in the copolymer structure significantly influenced the increase in the recorded capacitance current (*i*_c_) value. Furthermore, significantly higher *i*_c_ values were observed for the electrodes modified with the CNO–polymer hybrids (Figure 6b). Undoubtedly, the incorporation of the CNOs at the stage of material synthesis enhanced its electrochemical capacitance. The specific capacitance (*C*_s_) value was calculated for all samples from the integration of the CVs in the potential range from −600 to 0 mV vs Ag/AgCl (Table S16). The *C*_s_ value was calculated on the basis of the mass of the carbon dispersion deposited on the electrode surface, *m*, within the integration potential range, *ΔE*, according to the following equation:
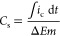
2As expected, an increase in the *C*_s_ values in the series from **C-1** (19 F g^–1^) to **C-4** (50 F g^–1^) was observed as a result of the increased average pore diameters and *S*_BET_ values (Table S16). The *C*_s_ values of the CNO–polymer hybrids (**C-(5-6)**) were more than twice as high as the values of the corresponding pyrolyzed pristine copolymers. Raymundo-Piñero et al. observed the important influence of micropores on the overall capacitance, leading to an enhanced *C*_s_.^59^ The dependence of the maximum double-layer capacitance on the electrode pore size was also reported when the pore size was between 1 and 5 nm.^60^ Therefore, the high degree of reversibility for double layers near the carbon film surface indicates that there are no chemical processes or other changes occurring between the charge and discharge cycles.

Figure 6c presents CVs of the GCE electrode modified with **C-6** in an ACN solution containing 0.1 M TBAPF_6_ recorded at different sweep rates (5–100 mV s^–1^). The capacitive current, *i*_c_, can also be expressed by the following equation:
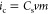
3The *i*_c_ values for **C-1**, **C-4**, **C-5**, and **C-6** depends linearly on the sweep rate at −200 mV vs Ag/AgCl (Figure 6d). The the *C*_s_ values of calculated by both methods, from the slopes of the *i*_*c*_*–v* plots and by integration of the *i*_c_*–E* curves, are consistent. These values are summarized in Table S16.

The **C-6** sample, which was chosen for a more in-depth electrochemical analysis, showed the highest value of *C*_s_ equal to 139 F g^–1^ (Table S16). No changes in the current for the GCE modified with **C-6** were observed after prolonged potential cycling between −1000 and +1000 mV vs Ag/AgCl (Figures S26 and S27). Multiscan CVs show that the film is stable; after 15 complete CV cycles, approximately 92% of the initial capacitance was preserved. The film exhibits stable and conductive behavior under CV conditions.

The effect of the supporting electrolyte on the electrochemical performance of **C-6** was determined (Figure S28). The CVs were recorded in an ACN solution containing different supporting electrolytes (TBAPF_6_, TBAP, TBA acetate, TBABF_4_, or TEAPF_6_) at a concentration of 0.1 M. In all cases, the *C*_s_ values are affected by their nature (Table S17), by the size of both the cations and the anions. The effects of the supporting electrolyte on the capacitances result from the structure of the double layer of the carbon materials and the degree of counterion penetration. Hence, an adequate pore size is very important to obtain high capacitance values.

## 4 Conclusion

In summary, we developed a family of nanostructured porous carbon materials by using CNOs and star polymers synthesized by RAFT polymerization. We used two approaches: (i) the attachment of presynthesized star copolymers to CNO directed by functional groups and defects on the surface of CNs and (ii) use of CNOs as a matrix for the controlled polymerization of the polymer chains on the surface of CNs. Thanks to the application of the controlled polymerization method and an appropriate selection of the length of the polymer chains, we have synthesized a series of hybrid porous materials with a specific pore size and homogeneous distribution.

Several experimental and theoretical methods were used to identify the correlations between the morphologies of the star polymers and their hybrids and the textural and electrochemical properties of the porous carbon materials. Our studies showed that, with the increasing length of the pore-forming polymeric chains, the textural characteristics of the carbon materials changed with increasing *S*_BET_, higher micro- and mesoporosity, and higher total pore volume. Furthermore, the use of CNOs in the preparation of carbonaceous materials resulted in the predominance of mesoporosity over microporosity and higher average pore size but lower *S*_BET_ and total pore volume. These textural changes affected the electrochemical properties of the carbon materials and assured easy penetration of the supporting electrolyte in the films. As expected, an increase in the *C*_s_ values in the series from **C-1** (19 F g^–1^) to **C-4** (50 F g^–1^) was observed as a result of the increased *S*_BET_ and average pore diameters. The *C*_s_ values of the pyrolyzed CNO–polymer hybrids were more than twice as high as the values of the corresponding annealed pristine star copolymers. The **C-6** sample showed the highest value of *C*_s_ equal to 139 F g^–1^. On consideration of the electrochemical performance, the method based on grafting the presynthesized polymers to the CNOs proved to be superior to the method involving the synthesis of polymers on the functionalized CNOs. However, the second approach resulted in more organized porous carbon structures. Importantly, according to our research, a very low content of the CNOs in the carbon materials (ca. 5 wt %) allows for a significant improvement in their structural and electrochemical properties.

## Supporting Information Available

^1^H and ^13^C NMR spectra of the chain transfer agent, ^1^H NMR spectra of all synthesized polymers, SEC traces of selected polymers, FTIR and Raman spectra of selected synthesized materials, images of suspensions of pristine CNO, **CNO-Br**, and **CNO-SC(S)OEt** in Et_2_O or toluene, XPS spectra of **C-1**, **C-5**, and **C-7**, HRTEM images of **C-1**, **C-4**, **C-5**, **C-6**, and **C-7**, CVs of GCE modified with **C-6**, electrochemical stability of GCE modified with **C-6**, details of all synthesis procedures, molecular weights and dispersity indexes obtained from ^1^H NMR and SEC analyses, Raman spectroscopy, surface elemental composition, chemical state, positions, fwhm, and relative area percentages of the deconvoluted C 1s and O 1s peaks obtained from XPS analyses of **C-1**, **C-4**, **C-5**, **C-6**, and **C-7** samples and the deconvoluted N 1s peaks of the **C-6** sample, TG results for selected polymers and carbon materials, specific capacitances of the polymer or hybrid materials after pyrolysis and specific capacitances of **C-6** for the different supporting electrolytes (PDF)
