# Supplementary material for: Polymeric Network Hierarchically Organized on Carbon Nano-onions: Block Polymerization as a Tool for the Controlled Formation of Specific Pore Diameters
Source: ACS Appl Polym Mater. 2022 Mar 17;4(4):2442–58. doi: 10.1021/acsapm.1c01788 (PMC9004317; doi:10.1021/acsapm.1c01788)
Supplement: Supplementary file 1 — ap1c01788_si_001.pdf [file ap1c01788_si_001.pdf]

Supporting Information

Polymeric Network Hierarchically Organized on Carbon Nano-Onions: Block Polymerization as a Tool for the Controlled Formation of Specific Pore Diameters

Gabriela Siemiaszko,[1]* Agnieszka Hryniewicka,*[1] *Joanna Breczko,*[1,2] *Olivia Fernandez Delgado,*[3] *Karolina H. Markiewicz,*[2] *Luis Echegoyen*[3] *and Marta E. Plonska-Brzezinska*[1]*

1. Department of Organic Chemistry, Faculty of Pharmacy with the Division of Laboratory Medicine, Medical University of Bialystok, Mickiewicza 2A, 15-222 Bialystok, Poland
2. Faculty of Chemistry, University of Bialystok, Ciolkowskiego 1K, 15-245 Bialystok, Poland
3. Department of Chemistry, University of Texas at El Paso, 500 W. University Ave., El Paso, TX 79968 USA

Correspondence: gsiemiaszko@gmail.com (G.S.); marta.plonska-brzezinska@umb.edu.pl (M.E.P.-B.)

Table of contents

Synthetic procedures

Synthesis of hexakis(S-methyl-O-ethyl dithiocarbonate)benzene (CTA)

General procedure for the synthesis of **6-*star*-(PMA)** polymers

Synthesis of 2-bromopropionyl CNO derivative (**CNO-Br**)

Synthesis of dithiocarbonate CNO derivative (**CNO-SC(S)OEt**)

Figure S1. ^{1}H NMR spectrum of chain transfer agent.

Figure S2. ^{13}C NMR spectrum of chain transfer agent.

Figure S3. ^{1}H NMR spectrum of **6-*star*-(PMA$_{25}$)**.

Figure S4. ^{1}H NMR spectrum of **6-*star*-(PMA$_{50}$)**.

Figure S5. ^{1}H NMR spectrum of **6-*star*-(PMA$_{100}$)**.

Figure S6. ^{1}H NMR spectrum of **6-*star*-(PMA$_{150}$)**.

Figure S7. ^{1}H NMR spectrum of **6-*star*-(PMA$_{25}$-*b*-PAS$_{200}$)**.

Figure S8. ^{1}H NMR spectrum of **6-*star*-(PMA$_{50}$-*b*-PAS$_{200}$)**.

Figure S9. ^{1}H NMR spectrum of **6-*star*-(PMA$_{100}$-*b*-PAS$_{200}$)**.

Figure S10. ^{1}H NMR spectrum of **6-*star*-(PMA$_{150}$-*b*-PAS$_{200}$)**.

Figure S11. ^{1}H NMR spectrum of **6-*star*-(PMA$_{25}$-*b*-PVPh$_{200}$)**.

Figure S12. ^{1}H NMR spectrum of **6-*star*-(PMA$_{50}$-*b*-PVPh$_{200}$)**.

Figure S13. ^{1}H NMR spectrum of **6-*star*-(PMA$_{100}$-*b*-PVPh$_{200}$)**.

Figure S14. ^{1}H NMR spectrum of **6-*star*-(PMA$_{150}$-*b*-PVPh$_{200}$)**.

Figure S15. ^{1}H NMR spectrum of **6-*star*-(PMA$_{25}$-*b*-PAS$_{200}$-N$_3$)**.

Figure S16. ^{1}H NMR spectrum of **6-*star*-(PMA$_{150}$-*b*-PAS$_{200}$-N$_3$)**.

Figure S17. SEC traces of **6-*star*-(PMA$_{100}$)** and **6-*star*-(PMA$_{100}$-*b*-PAS$_{200}$)**.

Figure S18. FTIR spectra of: (a) CTA, (b) **6-*star*-(PMA$_{150}$)**, (c) **6-*star*-(PMA$_{150}$-*b*-PAS$_{200}$)**, (d) **6-*star*-(PMA$_{150}$-*b*-PVPh$_{200}$)**, (e) **CP-4**, and (f) **C-4**.

Figure S19. FT-IR spectra of (a) **6-*star*-(PMA$_{150}$-*b*-PAS$_{200}$-N$_3$)**, (b) **6-*star*-(PMA$_{150}$-*b*-PAS$_{200}$)-CNO**, (c) **6-*star*-(PMA$_{150}$-*b*-PVPh$_{200}$)-CNO**, (d) **CP-6**, and (e) **C-6**.

Figure S20. Dispersity of pristine CNO, **CNO-Br**, and **CNO-SC(S)OEt** in diethyl ether or toluene due to ultrasonication for 1 h, after standing for 1 h or 72 h.

Figure S21. FT-IR spectra of (a) **CNO-Br**, (b) **CNO-SC(S)OEt**, (c) **CNO-PAS**, (d) **CNO-PVPh**, (e) **CP-7**, and (f) **C-7**.

Figure S22. Raman spectra of: (a) **6-*star*-(PMA_{150}-*b*-$PVPh_{200}$)**, (b) **6-*star*-(PMA_{150}-*b*-$PVPh_{200}$)-CNO**, (c) **C-4**, and (d) **C-6** (Panel A), and Panel B: (e) CNO, (f) **CNO-SC(S)OEt**, (g) **CNO-PAS**, and (h) **C-7**.

Figure S23. XPS spectra of the (A) C 1s and O 1s spectral region of **C-1**, (B) C 1s and O 1s spectral region of **C-5**, and (C) N 1s spectral region of **C-6**.

Figure S24. HRTEM images of **C-1** and **C-4**.

Figure S25. HRTEM images of **C-5**, **C-6**, and **C-7**.

Figure S26. CVs of GCE modified with **C-6** recorded at different potential ranges in ACN containing 0.1 M $TBAPF_6$ at a scan rate of 20 mV s^{-1}.

Figure S27. Electrochemical stability of GCE modified with **C-6** upon 15 cycles. CVs were recorded in ACN containing 0.1 M $TBAPF_6$ at a scan rate of 20 mV s^{-1}.

Figure S28. CVs of GCE modified with **C-6** recorded in ACN containing 0.1 M supporting electrolytes: (a) $TBAPF_6$, (b) TBA acetate, (c) $TBABF_4$, (d) TBAP, and (e) $TEAPF_6$ at a scan rate of 20 mV s^{-1}.

Table S1. Details of **6-*star*-(PMA)** synthesis procedures.

Table S2. Details of **6-*star*-(PMA-*b*-PAS)** synthesis procedures.

Table S3. Details of **6-*star*-(PMA-*b*-PVPh)** preparation procedures.

Table S4. Details of **CP-1**, **CP-2**, **CP-3**, and **CP-4** synthesis procedures.

Table S5. Details of **6-*star*-(PMA-*b*-PAS-N_3)** synthesis procedures.

Table S6. Details of **6-*star*-(PMA-*b*-PAS)-CNO** hybrids synthesis procedures.

Table S7. Details of the **6-*star*-(PMA-*b*-PVPh)-CNO** hybrids synthesis procedures.

Table S8. Details of **CP-5** and **CP-6** synthesis procedures.

Table S9. Summary of molecular weights and polydispersity indexes obtained from ^1H NMR and SEC analyses.

Table S10. Best-fit frequencies for the D and G bands obtained at 785 nm laser excitation energy and the relative I_D/I_G.

Table S11. Surface Elemental Composition of selected carbon samples determined by XPS.

Table S12. Chemical state, positions, FWHM, and relative area percentages of the deconvoluted C 1s peaks obtained from XPS analyses of **C-1**, **C-4**, **C-5**, **C-6**, and **C-7** samples.

Table S13. Chemical state, positions, FWHM, and relative area percentages of the deconvoluted O 1s peaks obtained from XPS analyses of **C-1**, **C-4**, **C-5**, **C-6**, and **C-7** samples.

Table S14. Chemical state, positions, FWHM, and relative area percentages of the deconvoluted N 1s peaks obtained from XPS analyses of **C-6**.

Table S15. TG results for selected polymers and carbon materials.

Table S16. Specific capacitances of the polymer or hybrid materials after pyrolysis, calculated from the CV studies.

Table S17. Specific capacitances of **C-6** for the different supporting electrolyte calculated from the CV studies.

Synthetic procedures

Synthesis of hexakis(S-methyl-O-ethyl dithiocarbonate)benzene (CTA)

KSCSOEt (9.0 equiv, 2.826 mmol, 453 mg) was added portionwise to a suspension of hexakis(bromomethyl)benzene (1.0 equiv, 0.314 mmol, 200 mg) in anhydrous THF (8.0 mL), followed by stirring for 24 h at room temperature (RT) in an Ar atmosphere. The precipitated salts were filtered from the reaction mixture using a fritted funnel and discarded. Water was added to the filtrate and extracted using AcOEt three times. The organic phase was separated, washed with an aqueous saturated NaCl solution, dried over $MgSO_4$, and filtered. The solvent was evaporated to give 300 mg of yellow oil. A crude mixture was purified using column chromatography on silica gel (AcOEt/hexane, gradient from 5-30%), affording the product as a white solid (189 mg, 67.8%). ^{1}H NMR ($CDCl_3$, 500 MHz): δ 4.66 (q, J = 7.1 Hz, 12 H, -OCH_2-), 4.44 (s, 12 H, -CH_2S-), 1.43 (t, J = 7.1 Hz, 18 H, -CH_3). ^{13}C NMR ($CDCl_3$, 126 MHz): δ 212.4, 135.6, 70.6, 35.7, 13.9. FTIR (neat): 2965, 1738, 1467, 1364, 1217, 1109, 1040, 444 cm^{-1}.

General procedure for the synthesis of 6-star-(PMA) polymers

CTA, methyl acrylate and AIBN were dissolved in anhydrous DMF. Ar was bubbled through this solution for 15 min. The reaction mixture was stirred for 24 h at 70 °C under Ar. The resulting solution of **6-*star*-(PMA$_{25}$)** or **6-*star*-(PMA$_{50}$)** was concentrated on a rotary evaporator, while **6-*star*-(PMA$_{100}$)** and **6-*star*-(PMA$_{150}$)** were precipitated with H_2O, followed by decanting the solvent and drying on a rotary evaporator. The resulting yellow sticky resins were vacuum dried (Table S1). **6-*star*-(PMA$_{25}$)**: ^{1}H NMR (DMSO-d_6, 500 MHz) characteristic signals: δ 4.62 (dd, J = 16.1, 6.1 Hz, 2H, OCH_2-), 3.57 (s, -OCH_3), 2.35-2.15 (m, -CH$_2$CHC(O)), 1.85-1.35 (m, CH_2CHC(O)-).

Synthesis of 2-bromopropionyl CNO derivative (CNO-Br)

The dispersion of CNOs (30.0 mg) with freshly powdered $AlCl_3$ (1.00 equiv, 4.60 mmol, 0.613 g) in anhydrous DCM (10.0 mL) was sonicated under Ar for 30 min. 2-Bromopropionyl bromide (2.06 mmol, 0.5 mL) was added dropwise at 0 °C, and stirring was continued at this temperature for 1 h, followed by stirring at RT for 18 h. The reaction mixture was then moved to the test tube, 20 mL of DCM was added, the suspension was sonicated for 10 min., and centrifuged for 10 min. The solvent was decanted from the black solid collected in the bottom of the test tube. The cycle of purification involving sonication, centrifugation, and decantation

was repeated several times using DCM and acetone sequentially. Finally, functionalized CNOs were dried under vacuum, affording 28.0 mg of a product as a black powder.

Synthesis of dithiocarbonate CNO derivative (CNO-SC(S)OEt)

2-Bromopropionyl-CNOs (CNO-Br, 20.0 mg) were suspended in anhydrous acetone (10.0 mL) under Ar and sonicated for 30 min. Next, KSC(S)OEt (3.12 mmol, 0.50 g) was added, and the reaction mixture was stirred for 18 h at RT. Acetone was then added, and the suspension was sonicated for 10 min and centrifuged, followed by decantation of the solvent. The purification procedure was repeated using acetone and MeOH multiple times, followed by drying in vacuo, affording 22.0 mg of the product as a black powder.

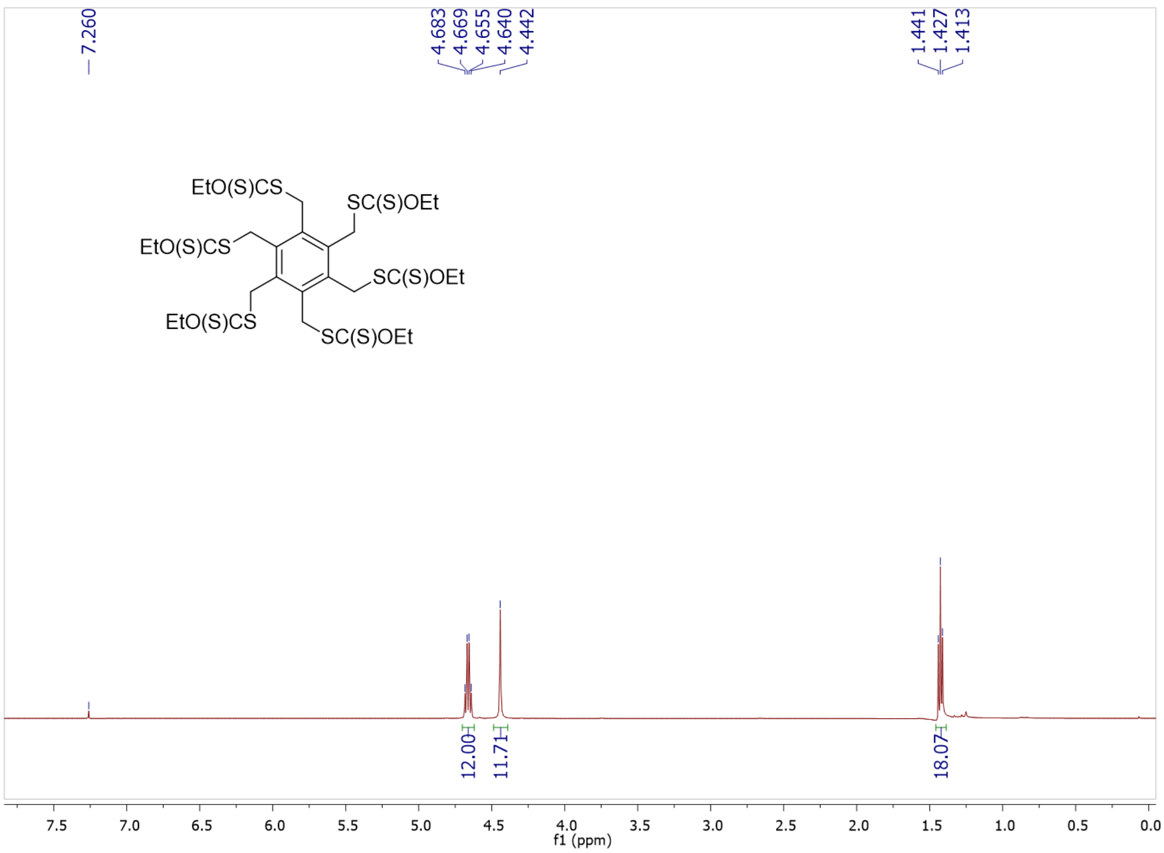

Figure S1. ^{1}H NMR spectrum of chain transfer agent.

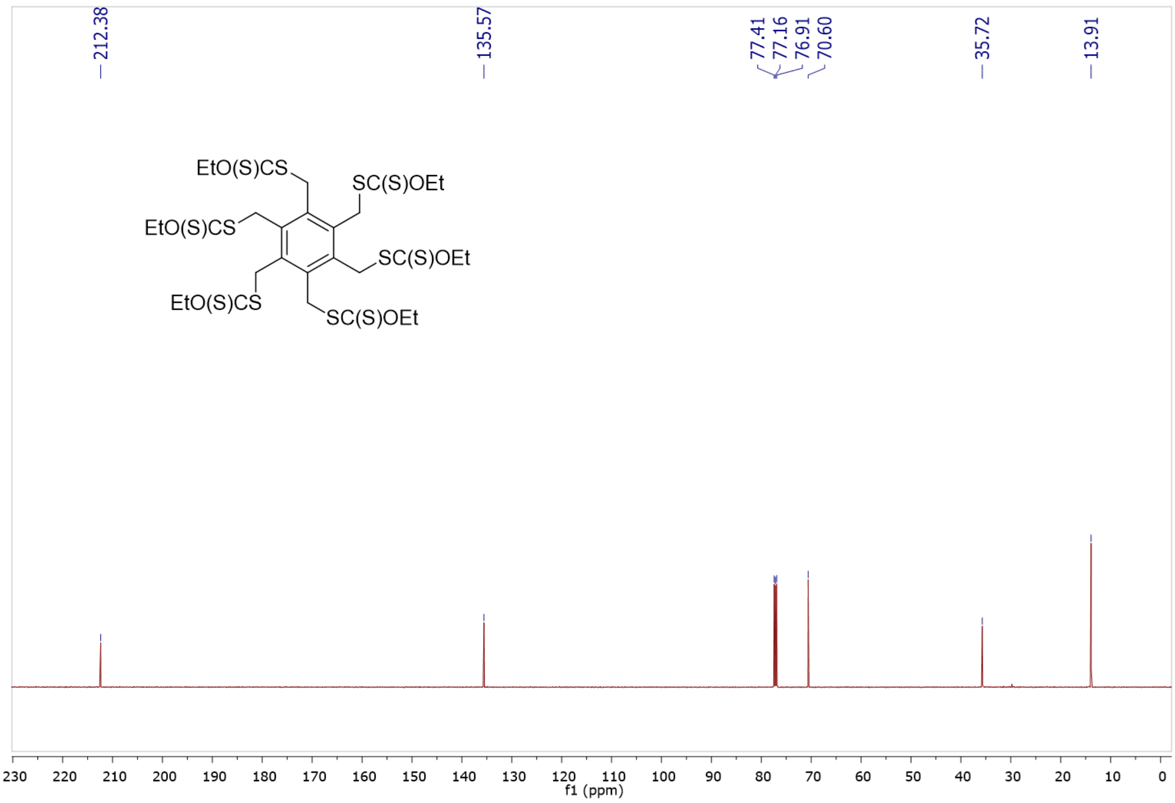

Figure S2. ^{13}C NMR spectrum of chain transfer agent.

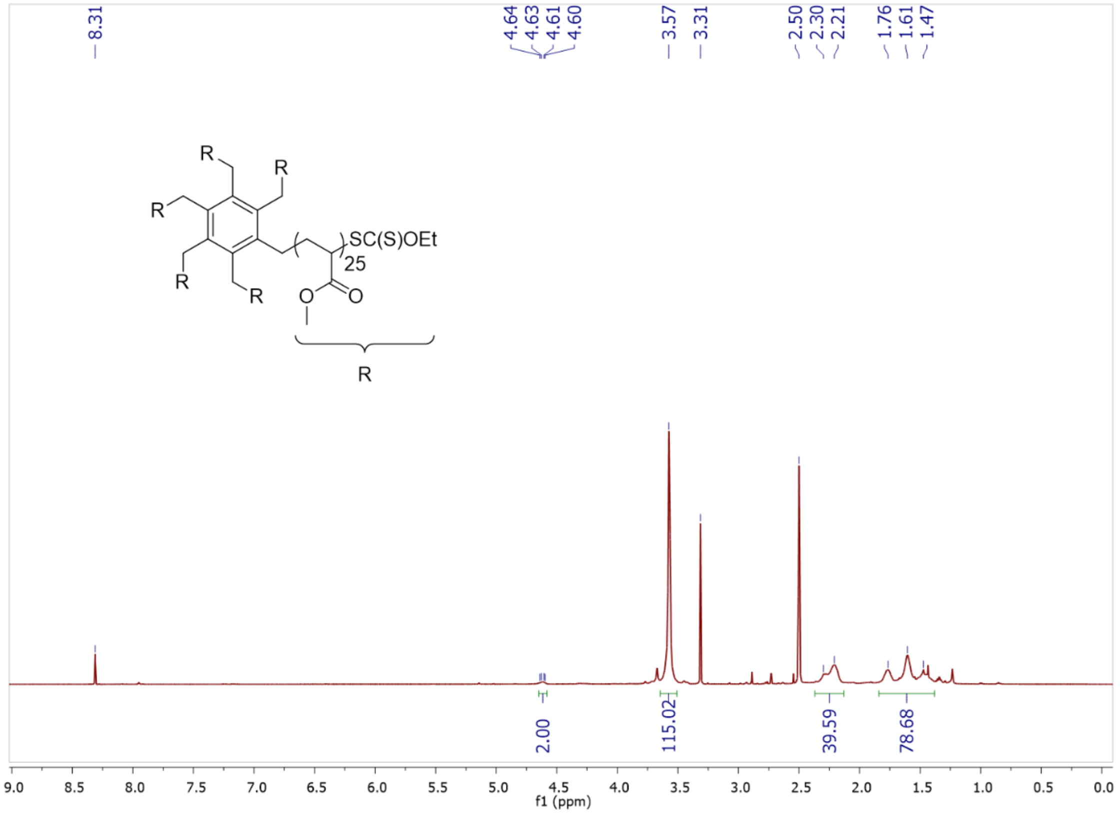

Figure S3. ^{1}H NMR spectrum of **6-*star*-(PMA$_{25}$)**.

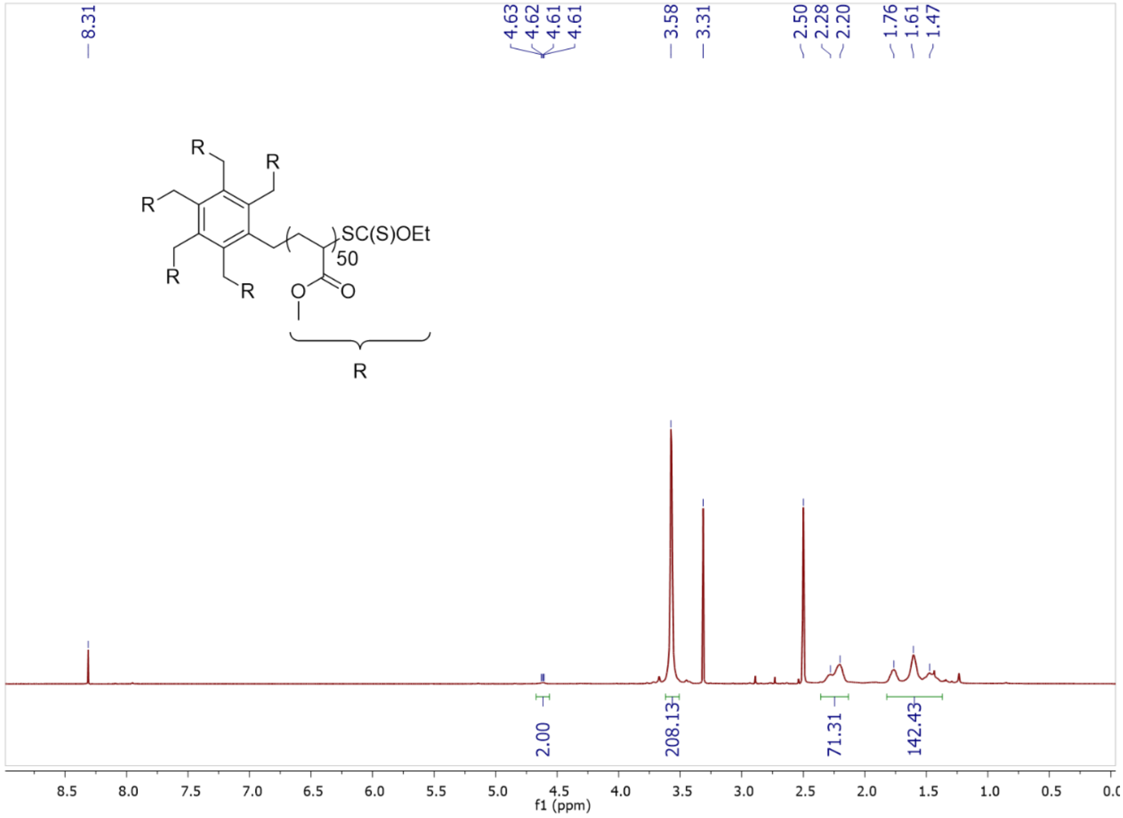

Figure S4. ^{1}H NMR spectrum of **6-*star*-(PMA$_{50}$)**.

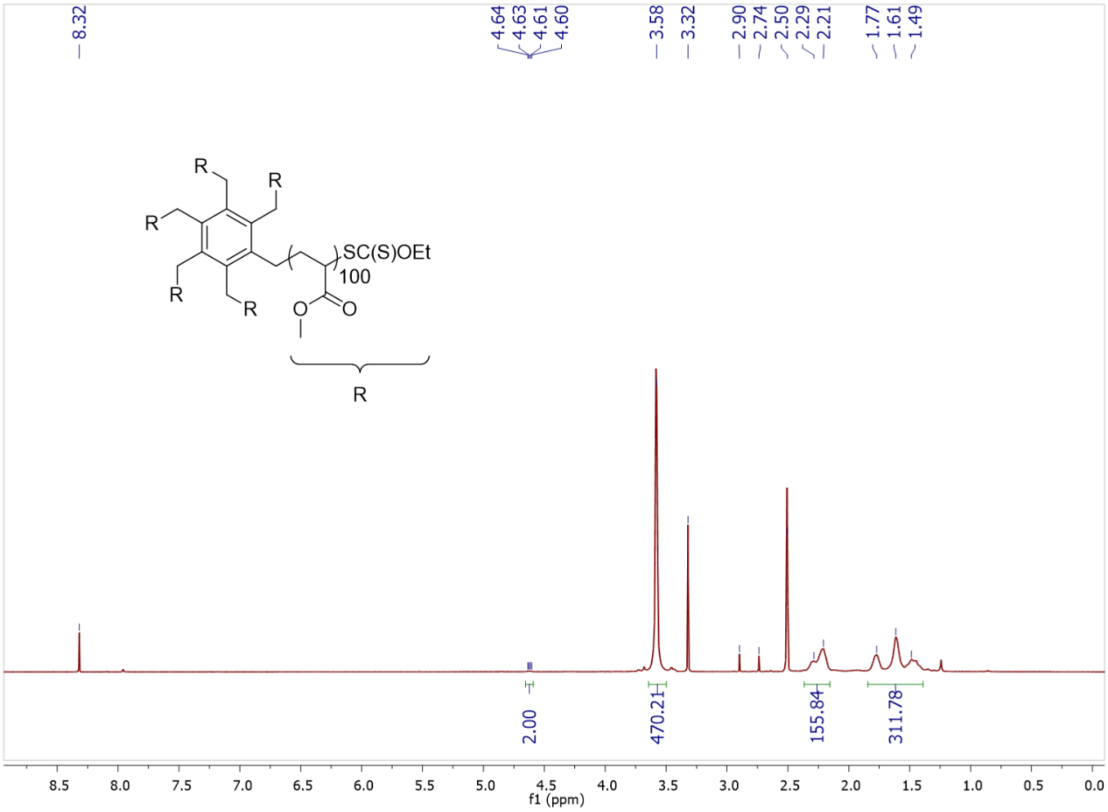

Figure S5. ^{1}H NMR spectrum of **6-*star*-(PMA$_{100}$)**.

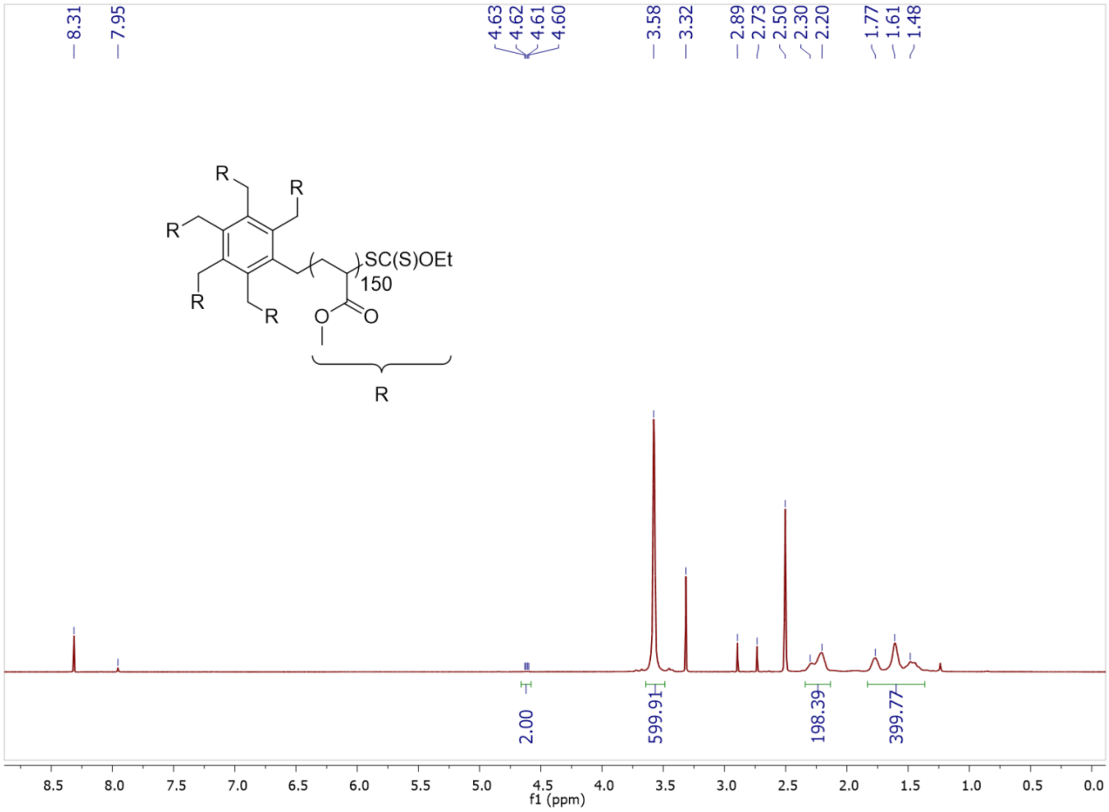

Figure S6. ^{1}H NMR spectrum of **6-*star*-(PMA$_{150}$)**.

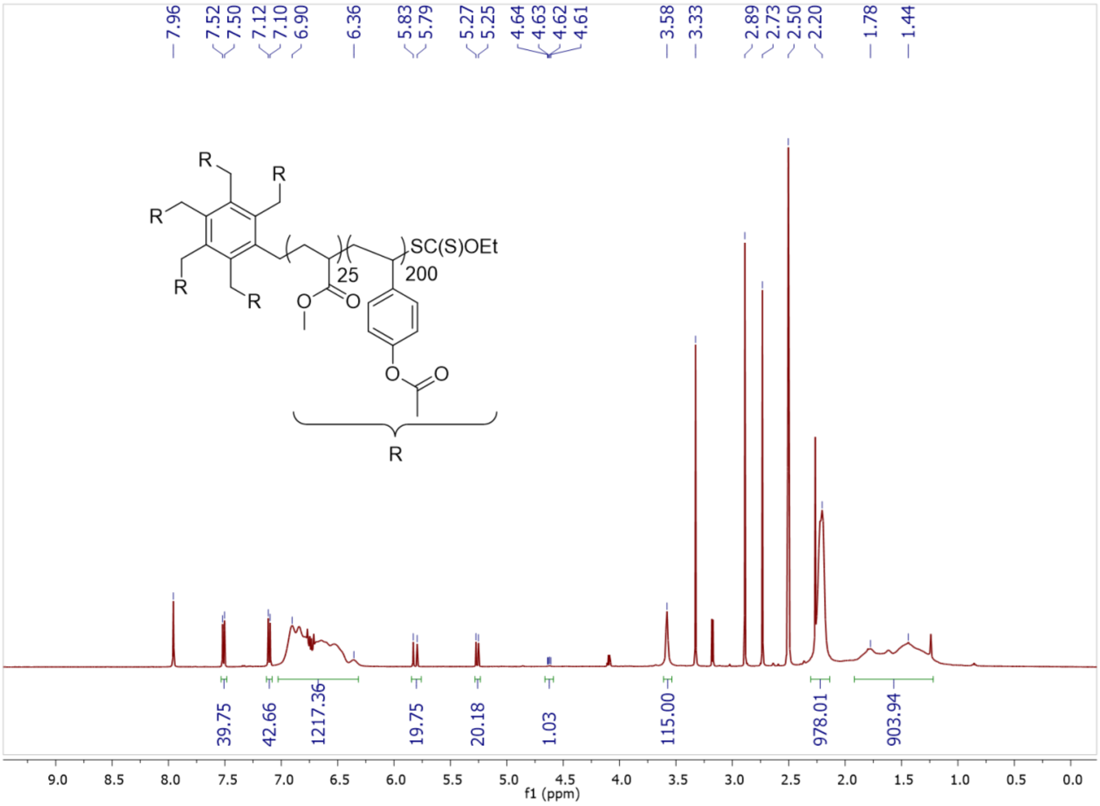

Figure S7. ¹H NMR spectrum of **6-*star*-(PMA$_{25}$-*b*-PAS$_{200}$)**.

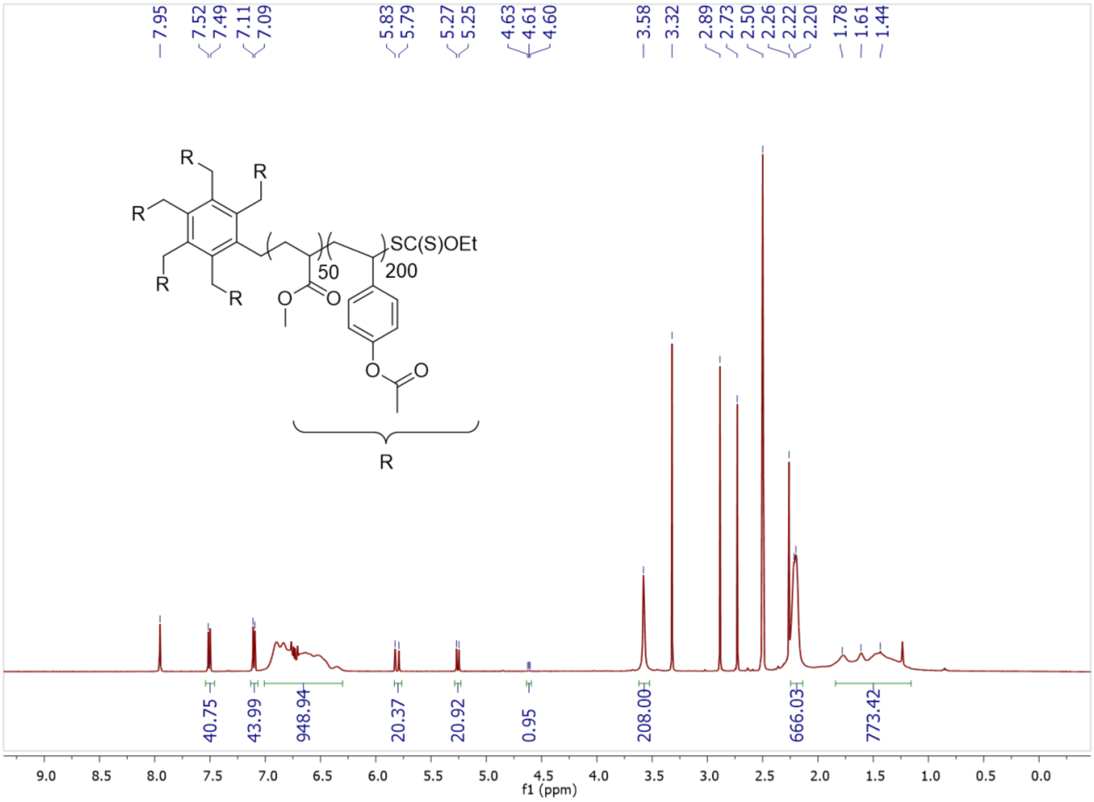

Figure S8. ¹H NMR spectrum of **6-*star*-(PMA$_{50}$-*b*-PAS$_{200}$)**.

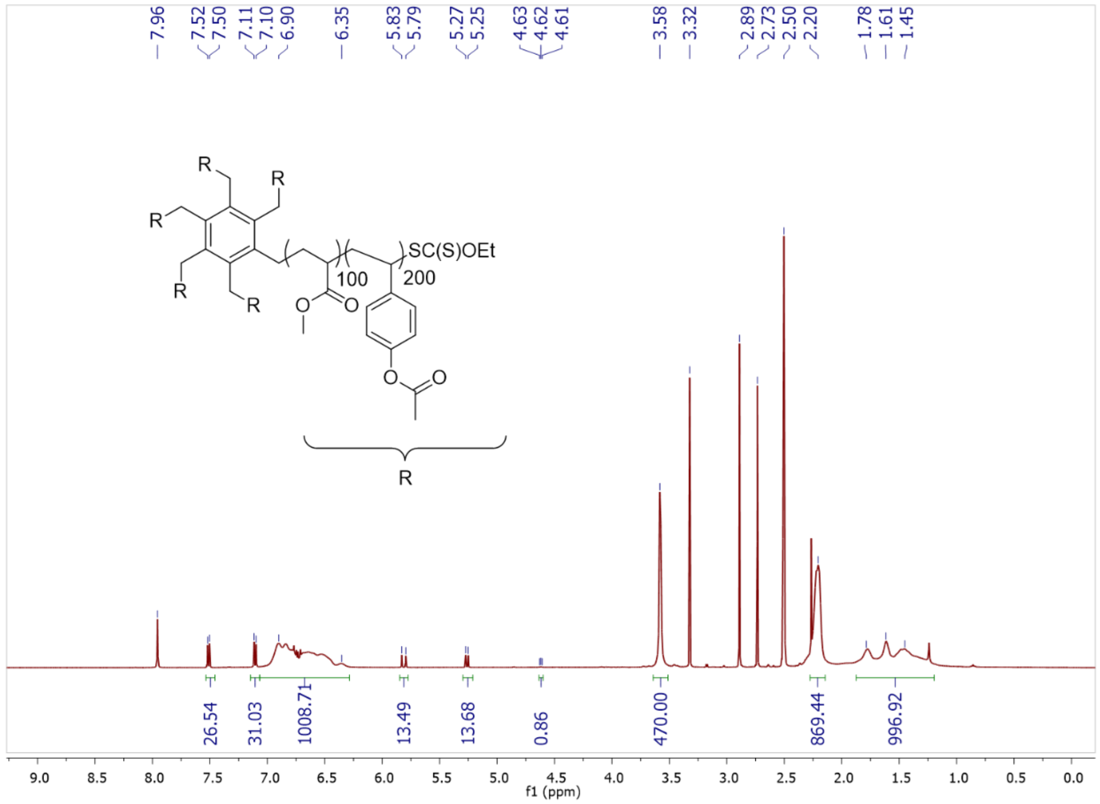

Figure S9. ^{1}H NMR spectrum of **6-*star*-(PMA$_{100}$-*b*-PAS$_{200}$)**.

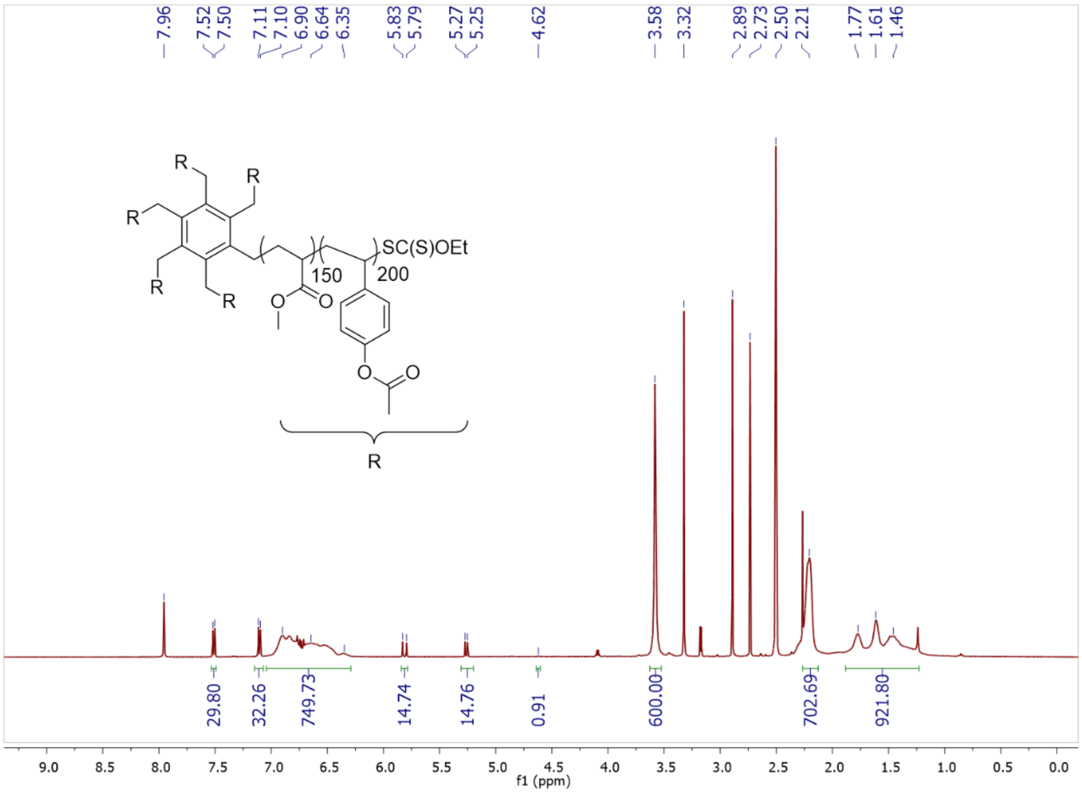

Figure S10. ^{1}H NMR spectrum of **6-*star*-(PMA$_{150}$-*b*-PAS$_{200}$)**.

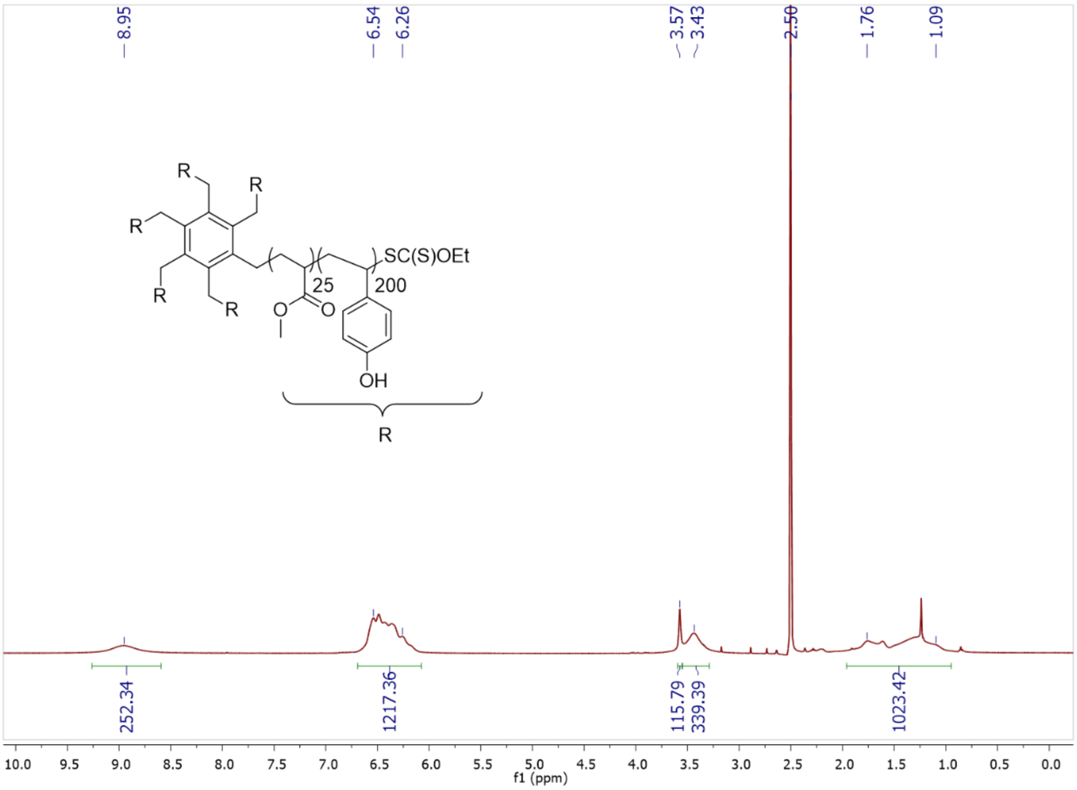

Figure S11. ^{1}H NMR spectrum of **6-*star*-(PMA$_{25}$-*b*-PVPh$_{200}$)**.

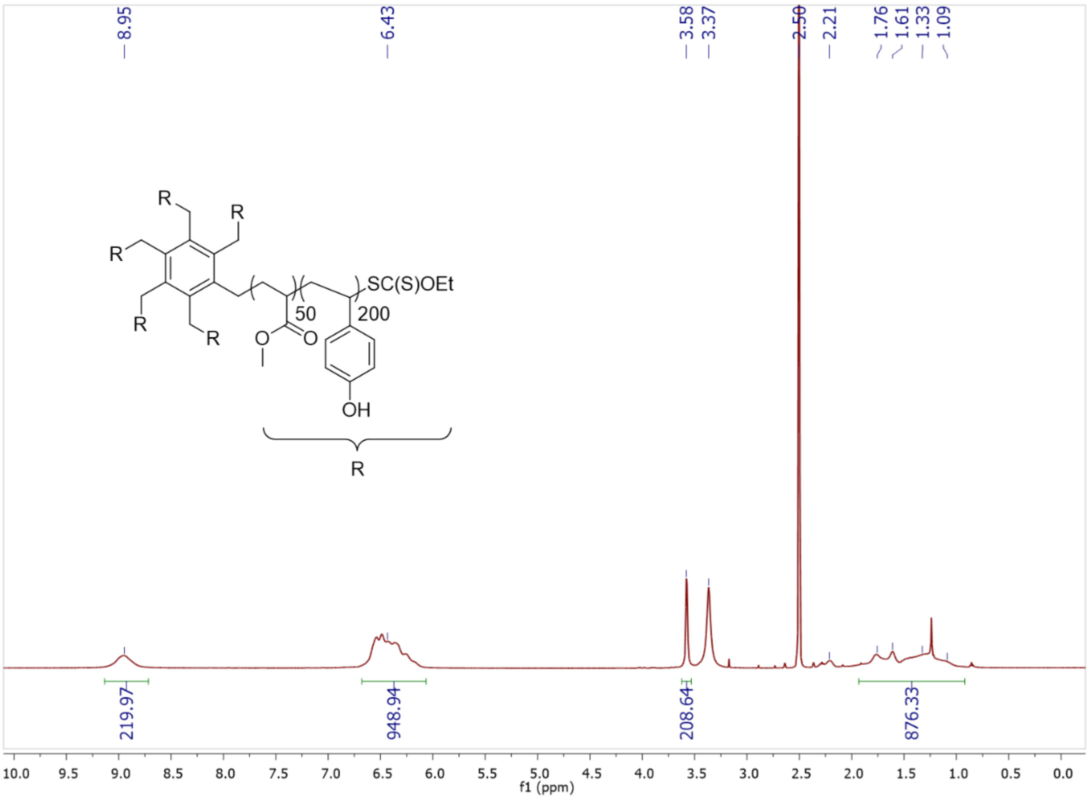

Figure S12. ^{1}H NMR spectrum of **6-*star*-(PMA$_{50}$-*b*-PVPh$_{200}$)**.

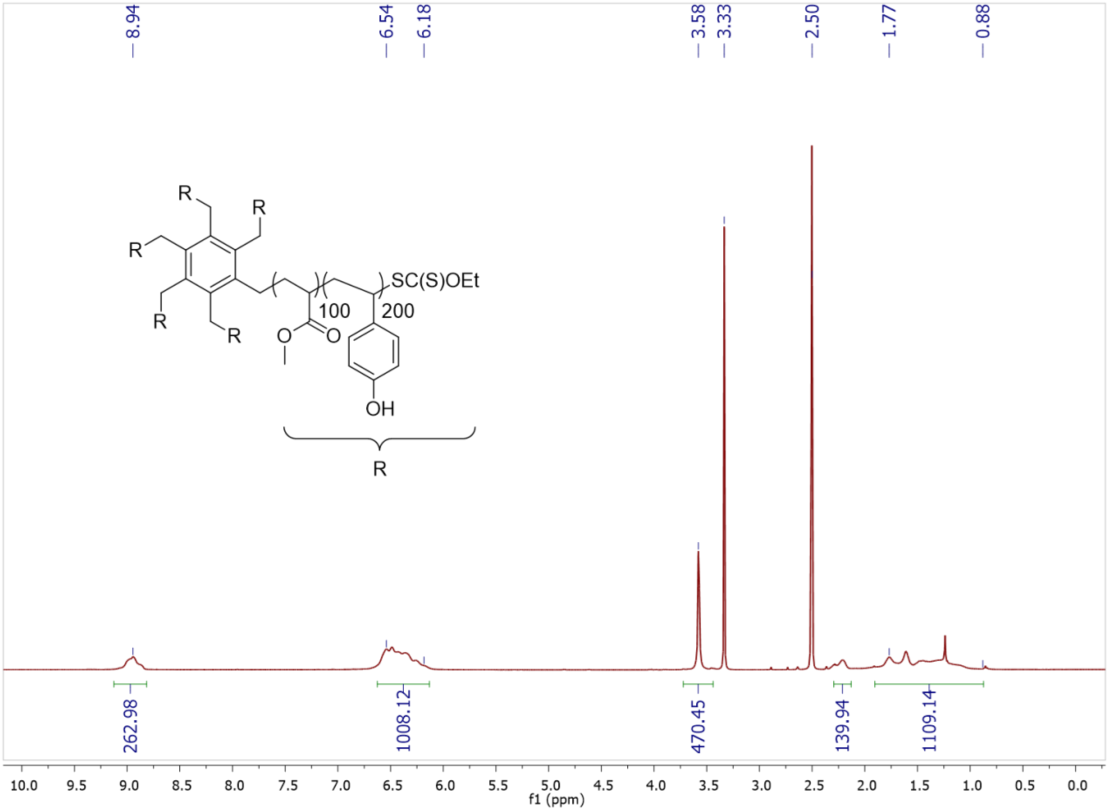

Figure S13. ^{1}H NMR spectrum of **6-*star*-(PMA$_{100}$-*b*-PVPh$_{200}$)**.

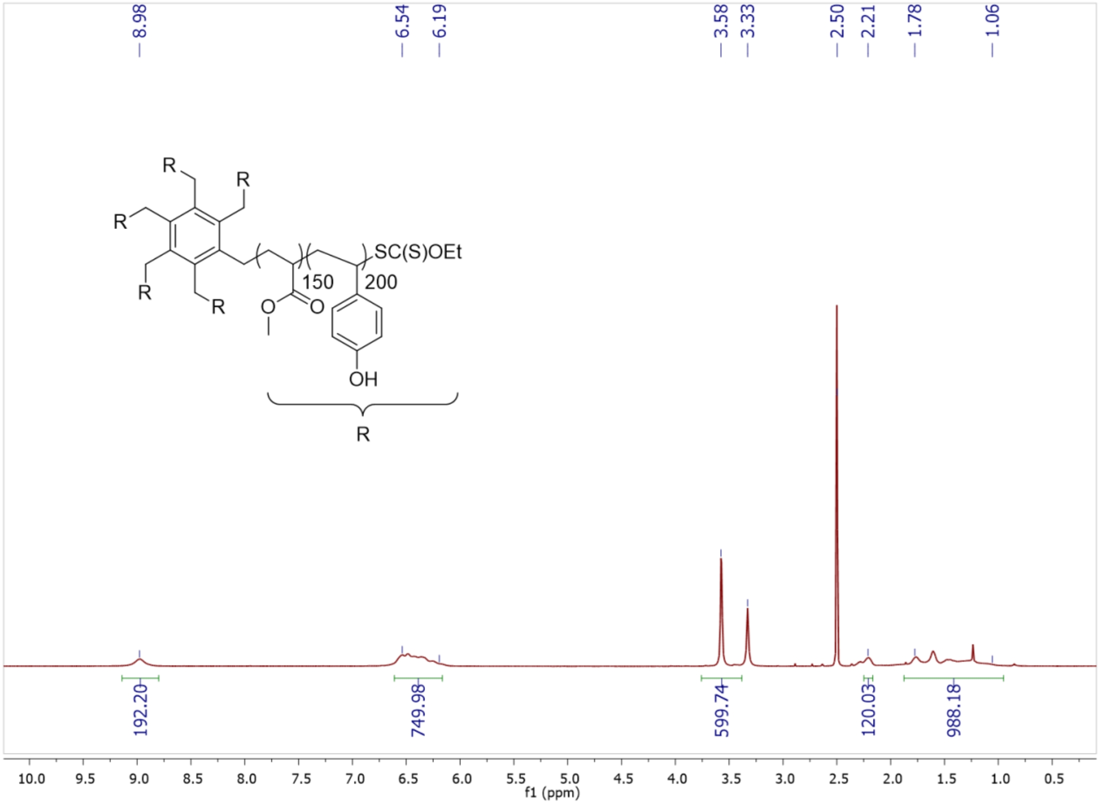

Figure S14. ^{1}H NMR spectrum of **6-*star*-(PMA$_{150}$-*b*-PVPh$_{200}$)**.

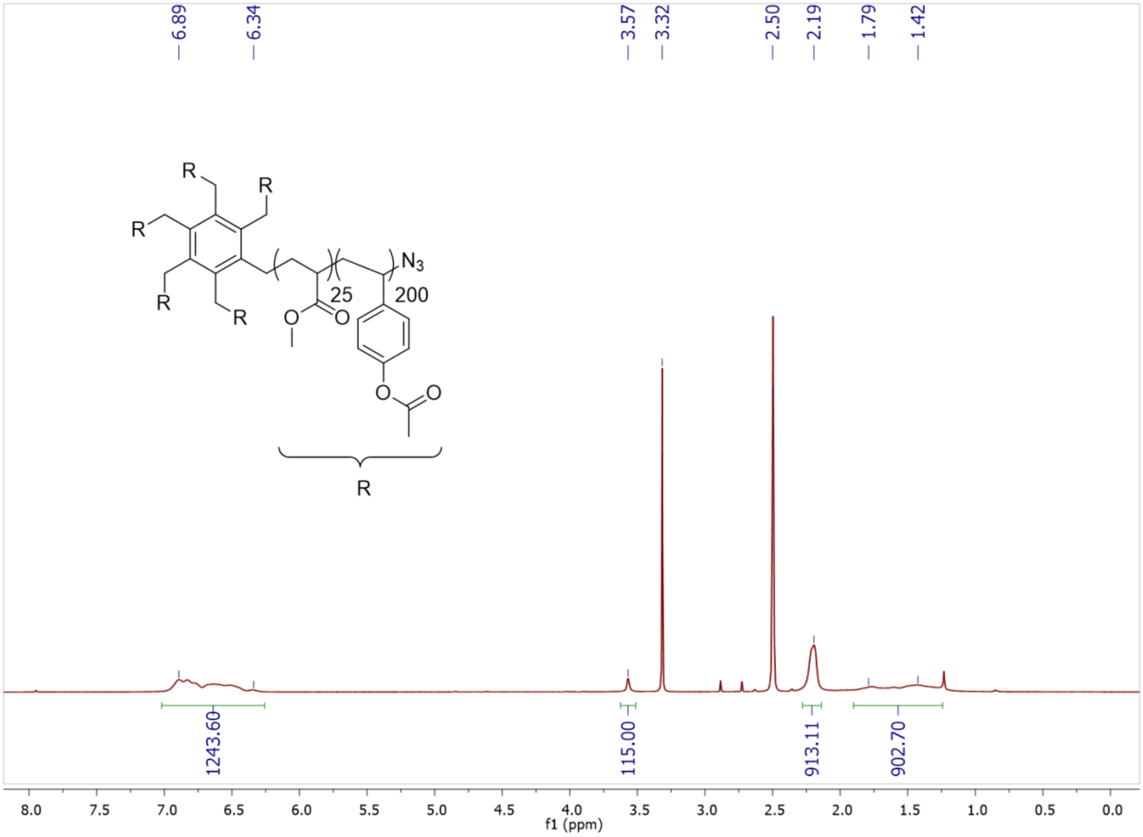

Figure S15. ^{1}H NMR spectrum of **6-*star*-(PMA$_{25}$-*b*-PAS$_{200}$-N$_3$)**.

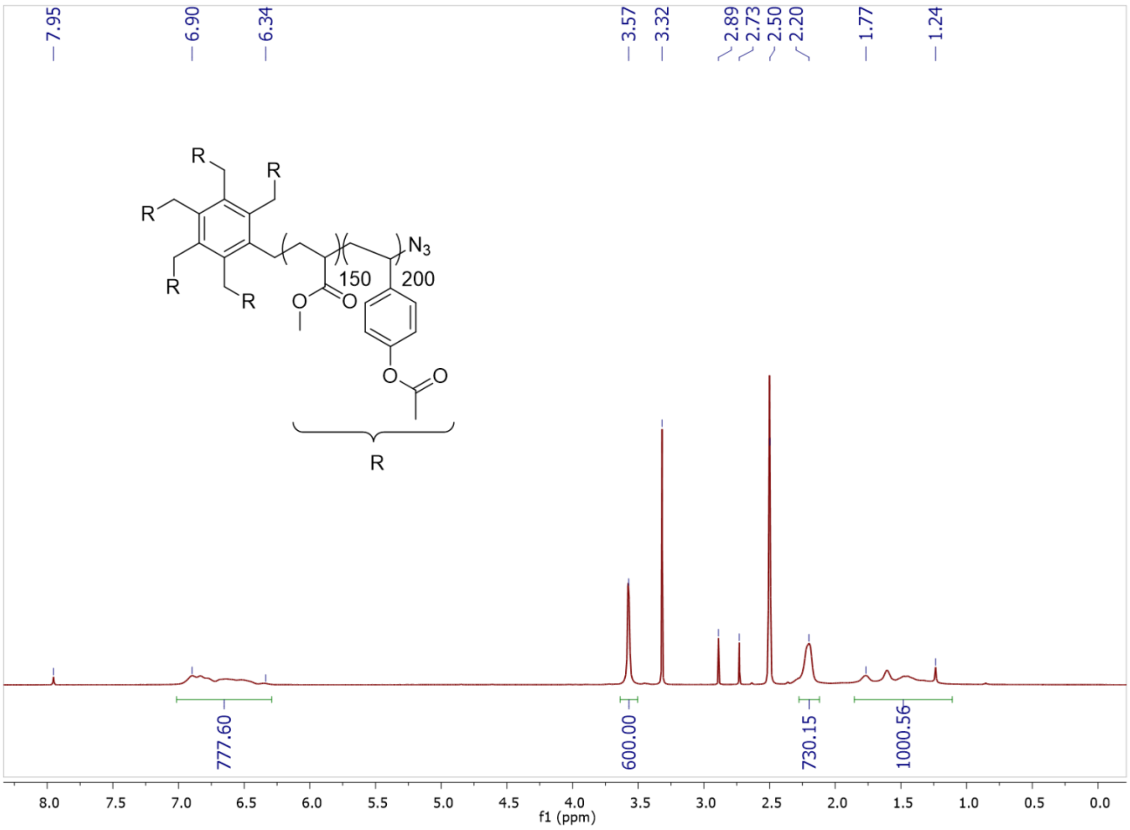

Figure S16. ^{1}H NMR spectrum of **6-*star*-(PMA$_{150}$-*b*-PAS$_{200}$-N$_3$)**.

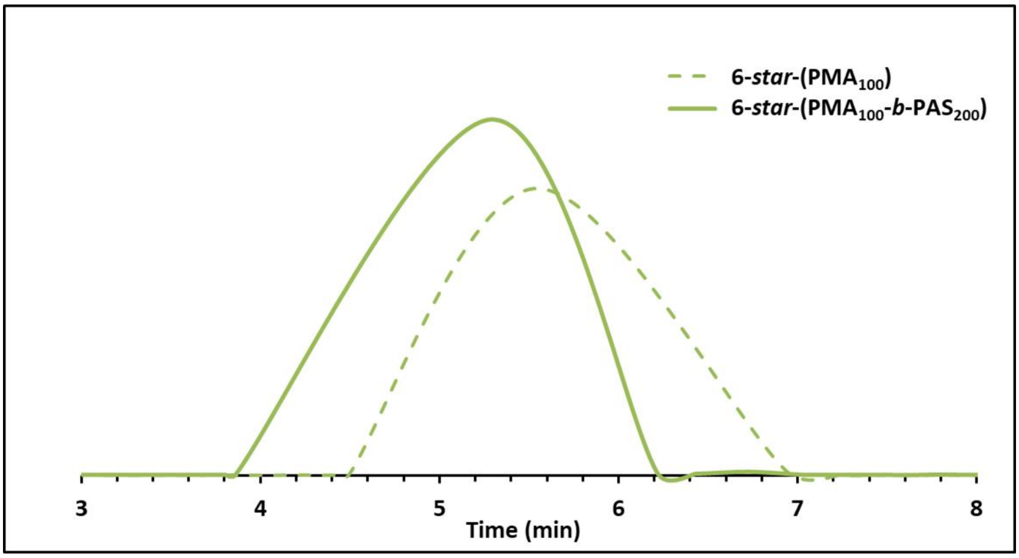

Figure S17. SEC traces of **6-*star*-(PMA_{100})** and **6-*star*-(PMA_{100}-*b*-PAS_{200})**.

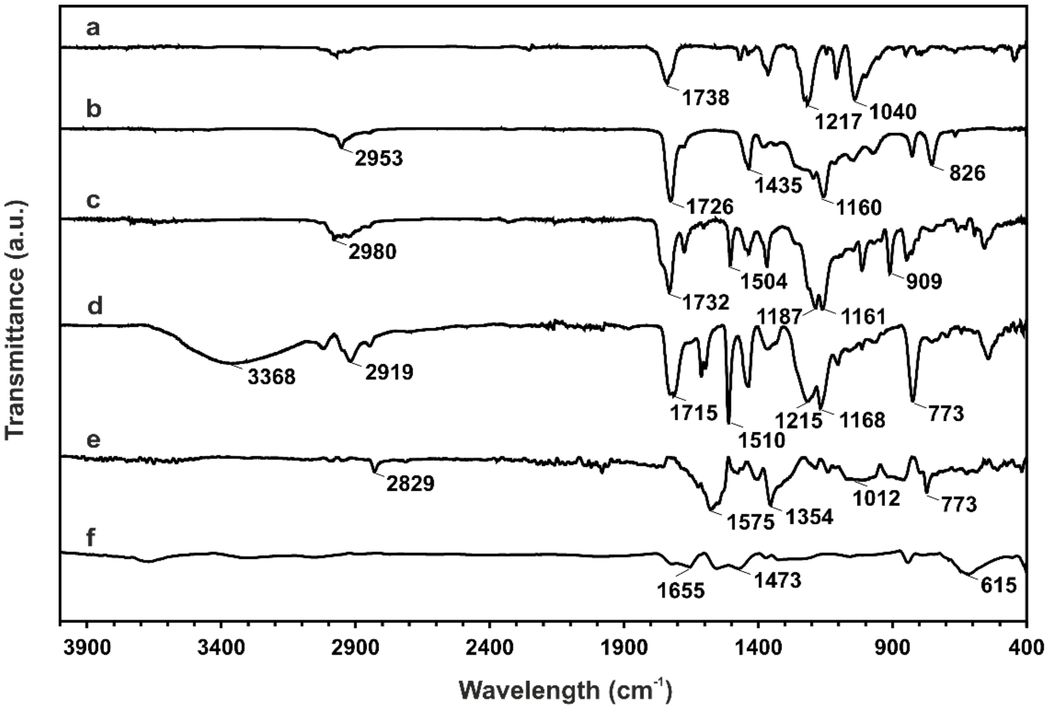

Figure S18. FTIR spectra of: (a) CTA, (b) **6-*star*-(PMA_{150})**, (c) **6-*star*-(PMA_{150}-*b*-PAS_{200})**, (d) **6-*star*-(PMA_{150}-*b*-$PVPh_{200}$)**, (e) **CP-4**, and (f) **C-4**.

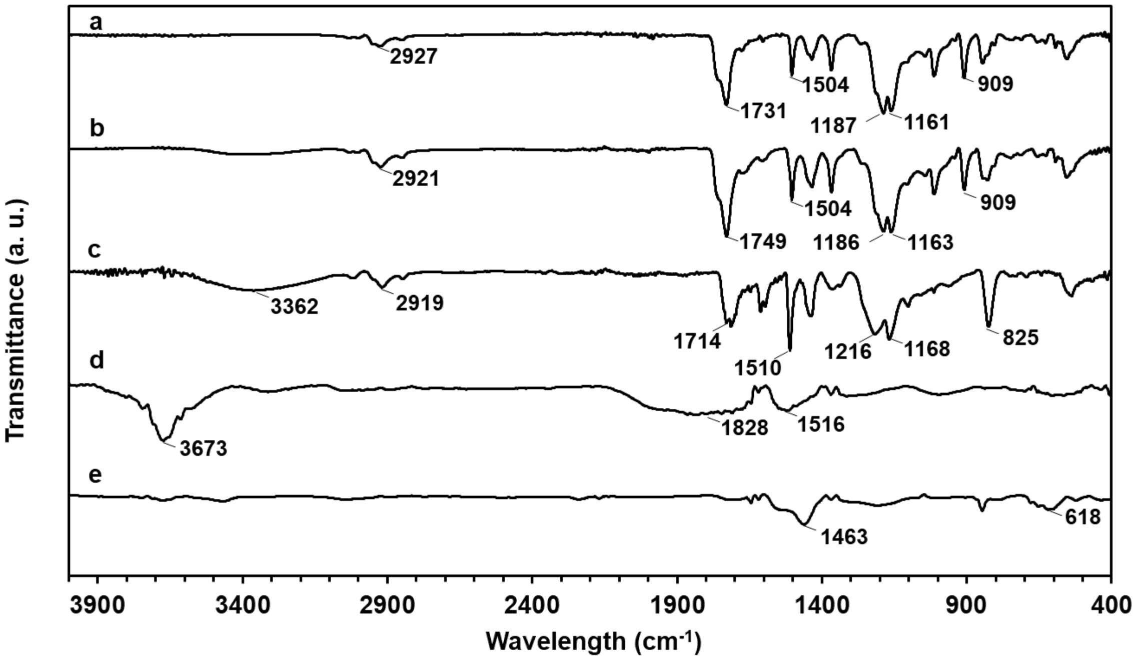

Figure S19. FT-IR spectra of (a) **6-*star*-(PMA_{150}-*b*-PAS_{200}-N_3)**, (b) **6-*star*-(PMA_{150}-*b*-PAS_{200})-CNO**, (c) **6-*star*-(PMA_{150}-*b*-$PVPh_{200}$)-CNO**, (d) **CP-6**, and (e) **C-6.**

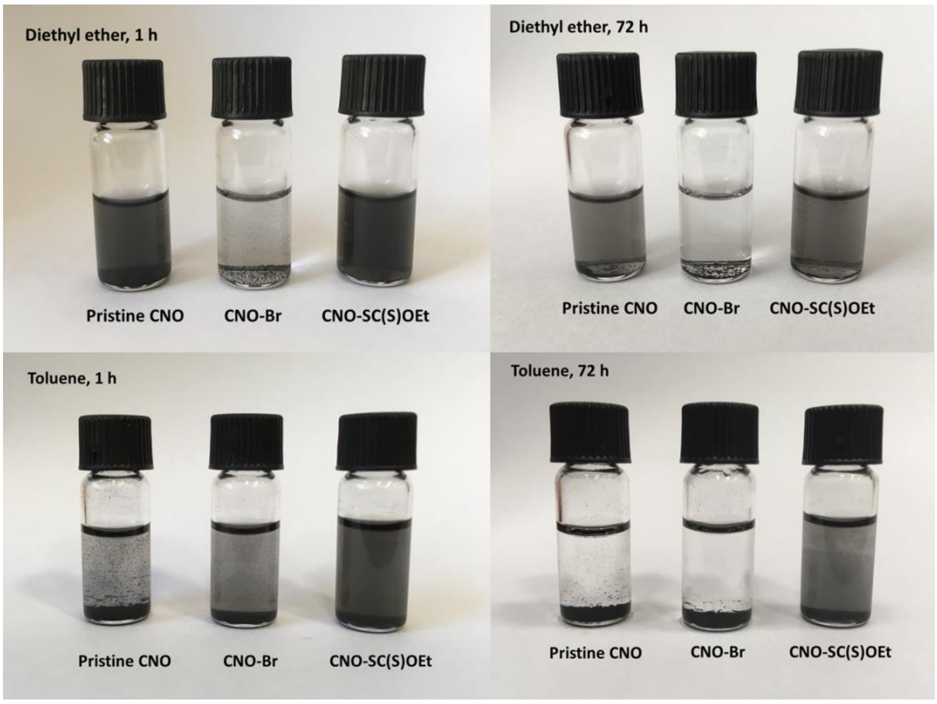

Figure S20. Dispersity of pristine CNO, **CNO-Br**, and **CNO-SC(S)OEt** in diethyl ether or toluene due to ultrasonication for 1 h, after standing for 1 h or 72 h.

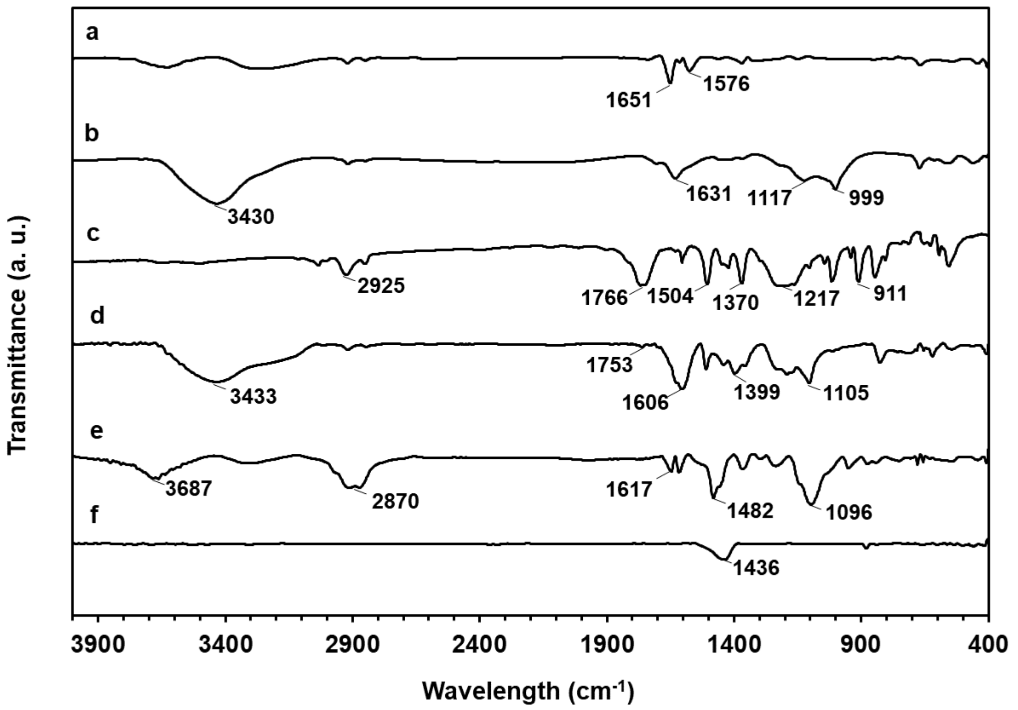

Figure S21. FT-IR spectra of (a) **CNO-Br**, (b) **CNO-SC(S)OEt**, (c) **CNO-PAS**, (d) **CNO-PVPh**, (e) **CP-7**, and (f) **C-7**.

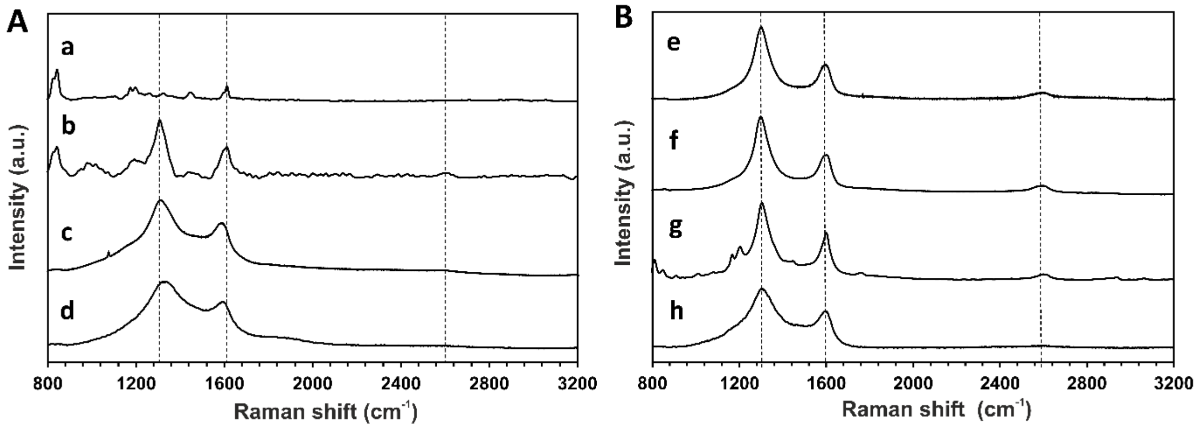

Figure S22. Raman spectra of: (a) **6-*star*-(PMA_{150}-*b*-$PVPh_{200}$)**, (b) **6-*star*-(PMA_{150}-*b*-$PVPh_{200}$)-CNO**, (c) **C-4**, and **(d) C-6** (Panel A), and Panel B: (e) **CNO**, (f) **CNO-SC(S)OEt**, (g) **CNO-PAS**, and (h) **C-7.**

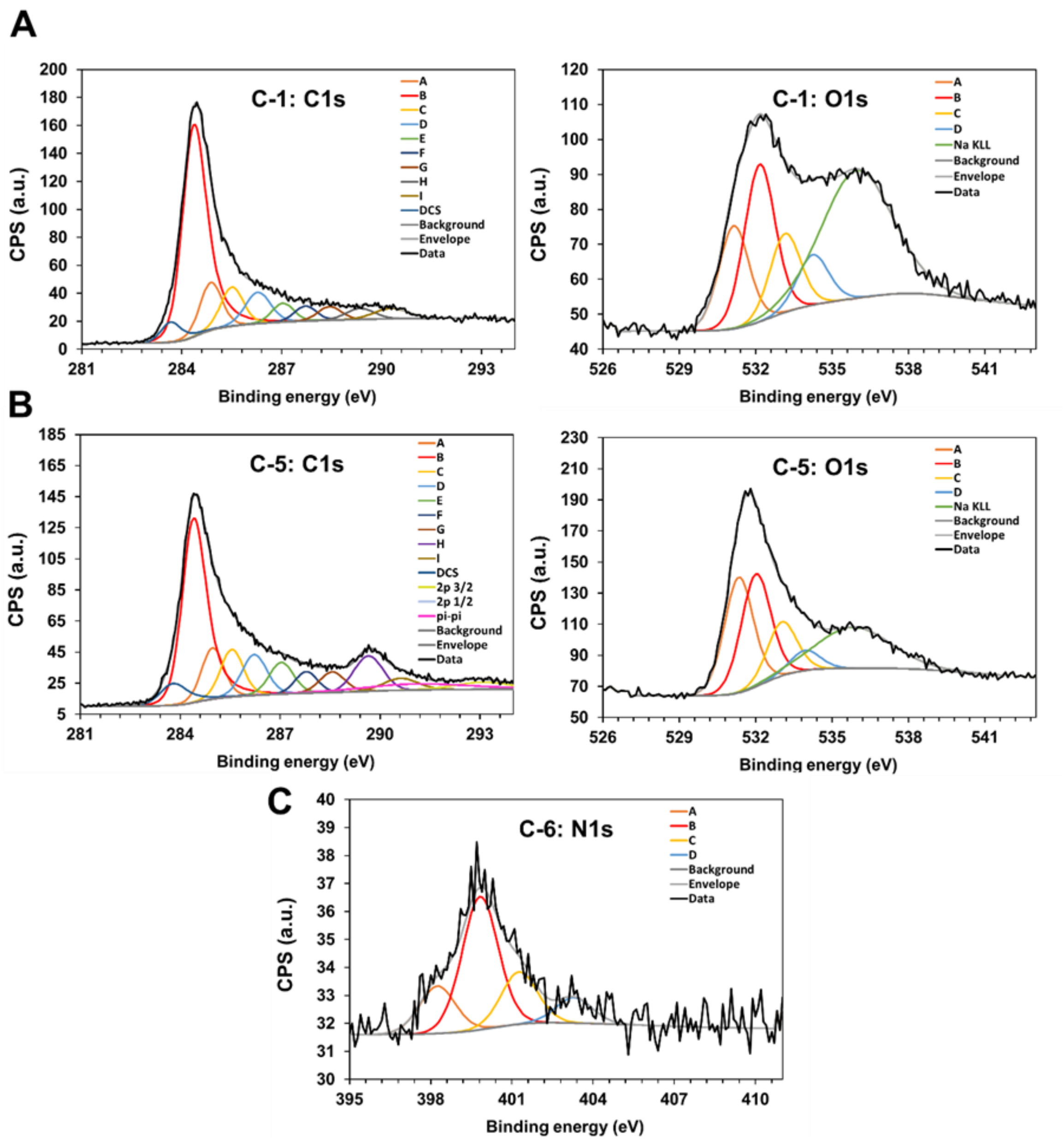

Figure S23. XPS spectra of the (A) C 1s and O 1s spectral region of **C-1**, (B) C 1s and O 1s spectral region of **C-5**, and (C) N 1s spectral region of **C-6**.

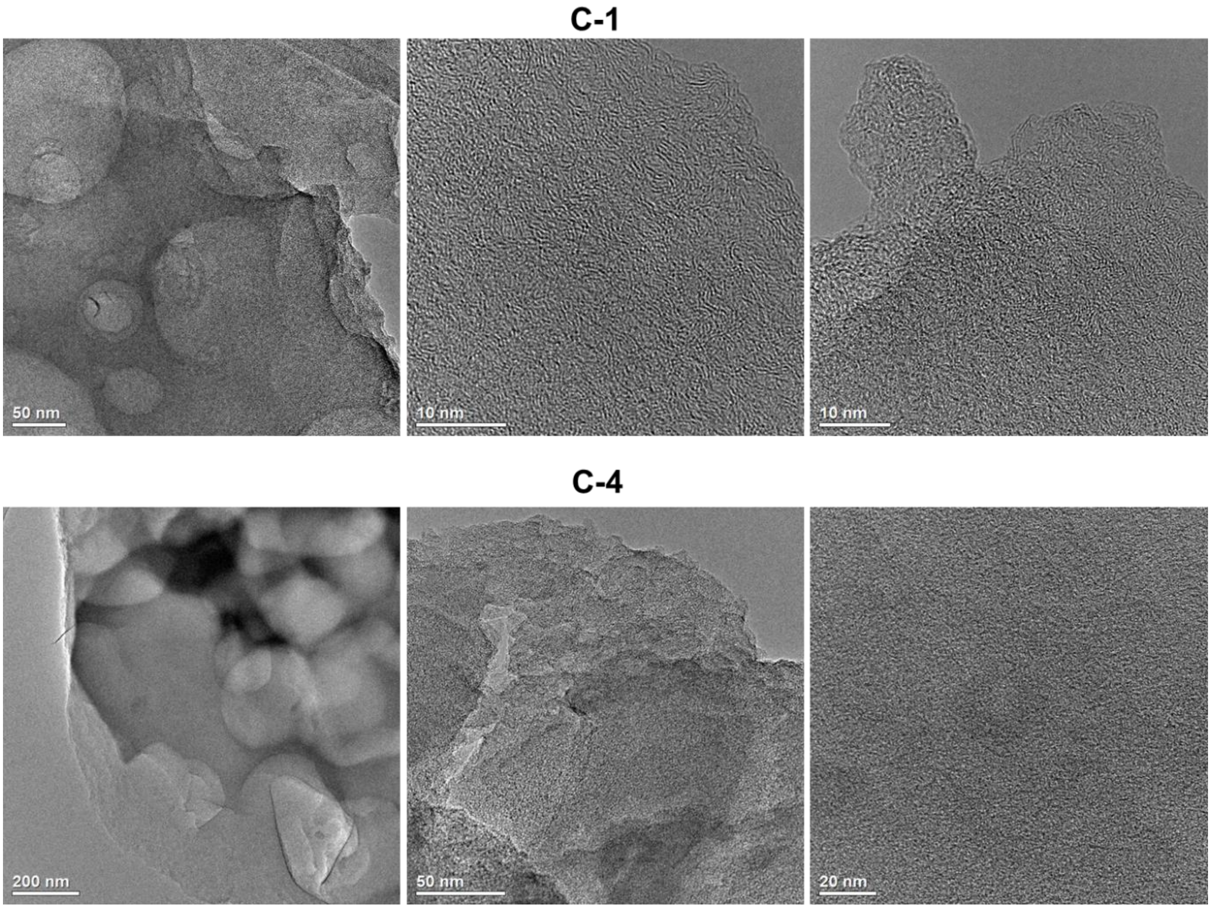

Figure S24. HRTEM images of **C-1** and **C-4**.

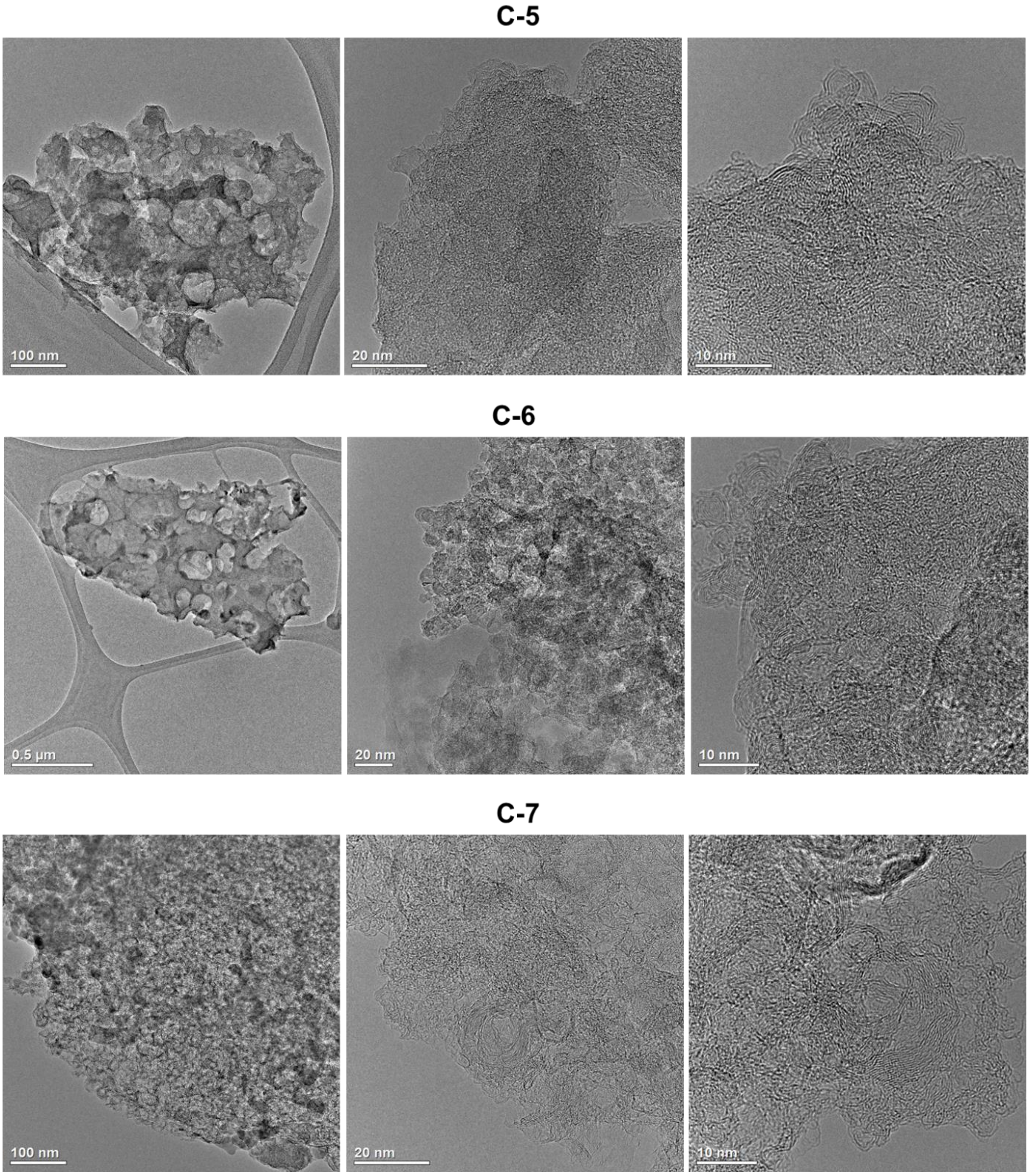

Figure S25. HRTEM images of **C-5**, **C-6**, and **C-7**.

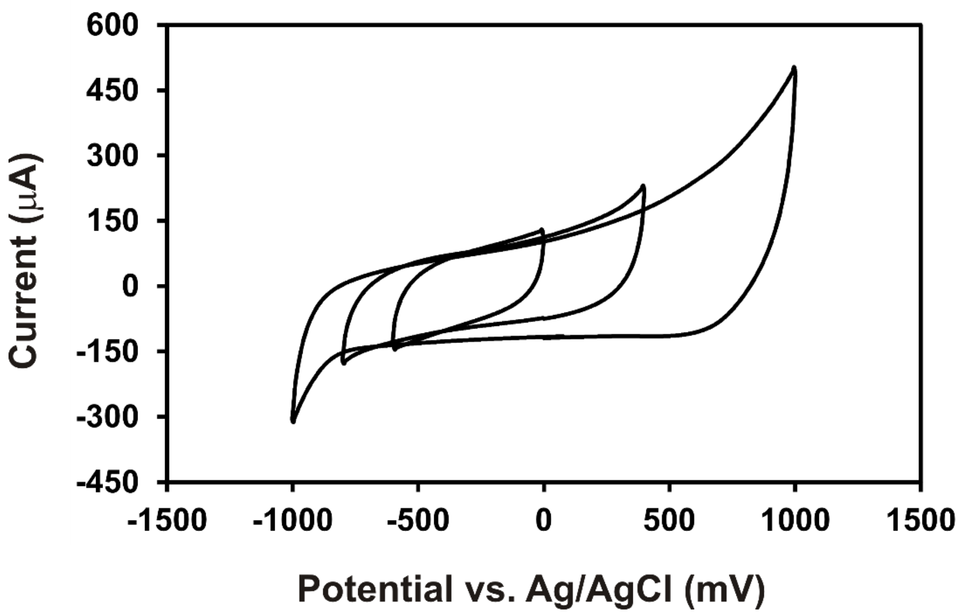

Figure S26. CVs of GCE modified with **C-6** recorded at different potential ranges in ACN containing 0.1 M $TBAPF_6$ at a scan rate of 20 mV s^{-1}.

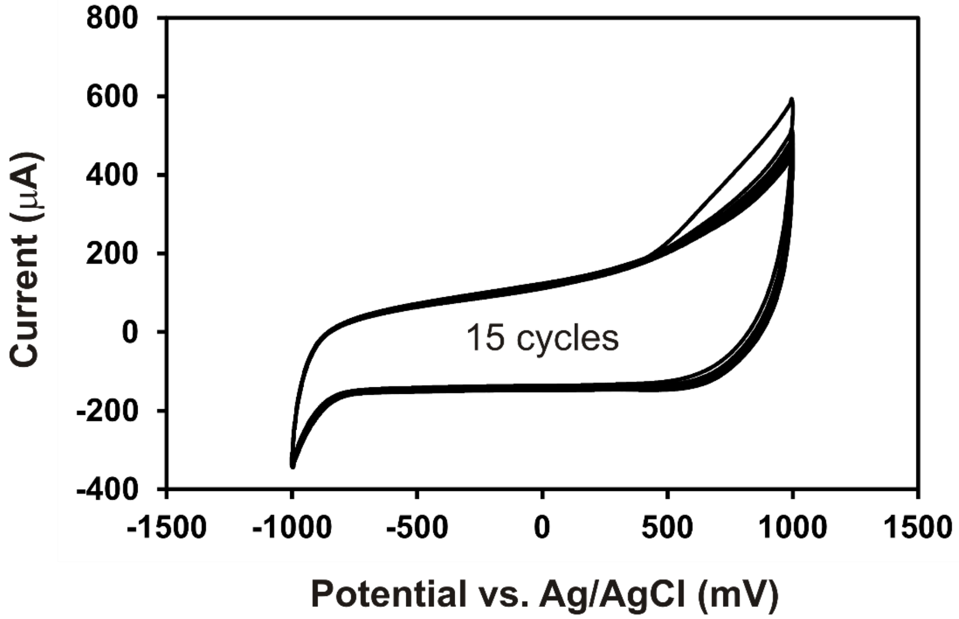

Figure S27. Electrochemical stability of GCE modified with **C-6** upon 15 cycles. CVs were recorded in ACN containing 0.1 M $TBAPF_6$ at a scan rate of 20 mV s^{-1}.

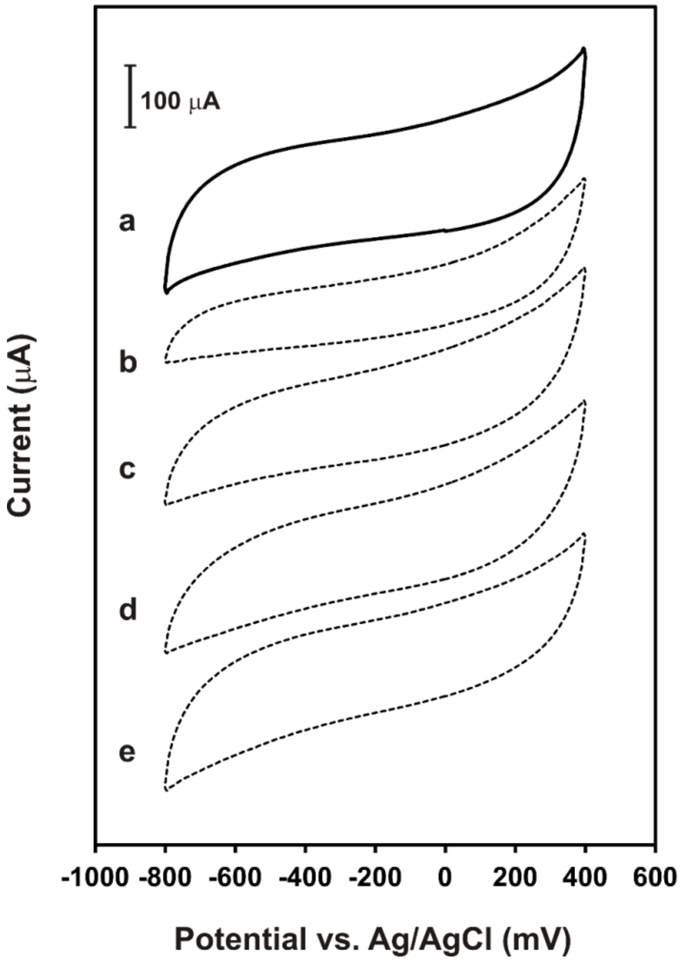

Figure S28. CVs of GCE modified with **C-6** recorded in ACN containing 0.1 M supporting electrolytes: (a) $TBAPF_6$, (b) TBA acetate, (c) $TBABF_4$, (d) TBAP, and (e) $TEAPF_6$ at a scan rate of 20 mV s^{-1}.

Table S1. Details of **6-*star*-(PMA)** synthesis procedures.

**Product**	**CTA**			**MA**			**AIBN**			**DMF (mL)**	**Product**				
	**equiv**	**n (mmol)**	**m (mg)**	**equiv**	**n (mmol)**	**V (mL)**	**equiv**	**n (mmol)**	**m (mg)**		**Mass (g)**	**$M_{n,\ th}$ (g mol^{-1})** [a]	**$M_{n,\ NMR}$ (g mol^{-1})**	**$M_{n,\ SEC}$ (g mol^{-1})**	**Đ**
**6-*star*-(PMA_{25})**	1.0	0.12	108.52	150.0	18.43	1.67	1.98	0.24	39.94	4.8	1.65	13 797	20 512	33 156	1.62
**6-*star*-(PMA_{50})**	1.0	0.11	93.48	300.0	31.75	2.88	1.98	0.21	34.41	8.2	2.71	26 710	36 525	65 329	1.66
**6-*star*-(PMA_{100})**	1.0	0.09	75.97	600.0	51.60	4.68	1.98	0.17	27.96	13.3	4.18	52 537	83 013	75 325	1.73
**6-*star*-(PMA_{150})**	1.0	0.07	58.60	900.0	59.61	5.40	1.98	0.13	21.57	15.4	5.17	78 364	104 191	131 368	1.73

[a] $M_{n,\ th} = M_{CTA} + M_{MA} \cdot equiv_{MA}$; the degree of monomer conversion was not taken into account.

Table S2. Details of **6-*star*-(PMA-*b*-PAS)** synthesis procedures.

**Product**	**6-*star*-(PMA)**					**AS**			**AIBN**			**DMF (mL)**	**Product**				
	**Substrate**	**$M_{n,\ th}$ (g mol^{-1})**	**equiv**	**n (mmol)**	**m (g)**	**equiv**	**n (mmol)**	**V (mL)**	**equiv**	**n (mmol)**	**m (mg)**		**Mass (g)**	**$M_{n,\ th}$** [a] **(g mol^{-1})**	**wt % PMA** [b]	**$M_{n,\ SEC}$ (g mol^{-1})**	**Đ**
**6-*star*-(PMA_{25}-*b*-PAS_{200})**	6-*star*-(PMA_{25})	13 797	1	0.019	0.266	1200	23.13	3.54	3.96	0.08	12.5	11.2	2.16	208 425	6.2	74 167	1.52
**6-*star*-(PMA_{50}-*b*-PAS_{200})**	6-*star*-(PMA_{50})	26 710	1	0.018	0.485	1200	21.77	3.33	3.96	0.08	11.8	10.6	1.98	221 338	13.4	124 171	1.43
**6-*star*-(PMA_{100}-*b*-PAS_{200})**	6-*star*-(PMA_{100})	52 537	1	0.016	0.853	1200	19.49	2.98	3.96	0.06	10.6	9.5	2.64	247 165	24.9	189 081	1.53
**6-*star*-(PMA_{150}-*b*-PAS_{200})**	6-*star*-(PMA_{150})	78 364	1	0.015	1.152	1200	17.64	2.70	3.96	0.06	9.56	8.6	1.82	272 992	36.2	215 766	1.58

[a] $M_{n,\ th} = M_{CTA} + M_{MA} \cdot equiv_{MA} + M_{AS} \cdot equiv_{AS}$; the degree of monomer conversion was not taken into account.
[b] wt % PMA = $(x_{MA} \cdot M_{MA})/(x_{MA} \cdot M_{MA} + x_{AS} \cdot M_{AS})$, where x_{MA} and x_{AS} are integrated signals taken from ^{1}H NMR spectrum; x_{MA} was defined by integrating singlet from 3 H of -CH_3 group at 3.59 ppm, while x_{AS} corresponds to integrated multiplet at approx. 6.2 - 6.6 ppm from 4 H of aromatic ring.

Table S3. Details of **6-*star*-(PMA-*b*-PVPh)** preparation procedures.

Product	6-*star*-(PMA-*b*-PAS)					0.5 M KOH/MeOH (mL)	MeOH (mL)	Product	
	Substrate	$M_{n,\ th}$ (g mol^{-1})	equiv	n (mmol)	m (g)			Mass (g)	wt % PMA [a]
**6-*star*-(PMA_{25}-*b*-$PVPh_{200}$)**	6-*star*-(PMA_{25}-*b*-PAS_{200})	208 425	1	0.004	0.900	3.0	3.0	0.580	10.9
**6-*star*-(PMA_{50}-*b*-$PVPh_{200}$)**	6-*star*-(PMA_{50}-*b*-PAS_{200})	221 338	1	0.004	0.900	3.0	3.0	0.646	17.7
**6-*star*-(PMA_{100}-*b*-$PVPh_{200}$)**	6-*star*-(PMA_{100}-*b*-PAS_{200})	247 165	1	0.004	0.900	3.0	3.0	0.588	29.3
**6-*star*-(PMA_{150}-*b*-$PVPh_{200}$)**	6-*star*-(PMA_{150}-*b*-PAS_{200})	272 992	1	0.003	0.900	3.0	3.0	0.636	40.5

[a] wt % PMA = $(x_{MA} \cdot M_{MA})/(x_{MA} \cdot M_{MA} + x_{VPh} \cdot M_{VPh})$, where x_{MA} and x_{VPh} are integrated signals taken from appropriate ^{1}H NMR spectrum; x_{MA} was defined by integrating singlet from 3 H of -CH_3 group at 3.59 ppm, while x_{VPh} corresponds to integrated multiplet at approx. 6.2 – 6.6 ppm from 4 H of aromatic ring.

Table S4. Details of **CP-1**, **CP-2**, **CP-3**, and **CP-4** synthesis procedures.

Product	6-*star*-(PMA-*b*-PVPh)					HCHO						10% NaOH (mL)	Product (g)
						1st portion			2nd portion				
	Substrate	$M_{n,\ th}$ (g mol^{-1})	equiv	m (g)	n (mmol)	equiv	n HCHO (mol)	V (mL)	equiv	n (mol)	V (mL)		
**CP-1**	6-*star*-(PMA_{25}-*b*-$PVPh_{200}$)	157 977	1	0.500	0.003	1.3	0.004	0.283	5.0	0.015	1.131	2.7	0.769
**CP-2**	6-*star*-(PMA_{50}-*b*-$PVPh_{200}$)	170 890	1	0.550	0.003	1.3	0.004	0.288	5.0	0.015	1.150	2.8	0.521
**CP-3**	6-*star*-(PMA_{100}-*b*-$PVPh_{200}$)	196 717	1	0.500	0.003	1.0	0.003	0.227	4.0	0.012	0.908	2.2	0.670
**CP-4**	6-*star*-(PMA_{150}-*b*-$PVPh_{200}$)	222 544	1	0.550	0.003	1.0	0.003	0.221	4.0	0.012	0.883	2.1	0.624

Table S5. Details of **6-*star*-(PMA-*b*-PAS-N_3)** synthesis procedures.

Product	6-*star*-(PMA-*b*-PAS)					$EtSO_2N_3$			AIBN			DMF (mL)	Product	
	Substrate	$M_{n, th}$ (g mol^{-1})	equiv	n (mmol)	m (g)	equiv	n (mmol)	m (mg)	equiv	n (mmol)	m (mg)		Mass (g)	$M_{n, th}$ (g mol^{-1})
**6-*star*-(PMA$_{25}$-*b*-PAS$_{200}$-N_3)**	6-*star*-(PMA$_{25}$-*b*-PAS$_{200}$)	208 425	1	0.004	0.8	197.9	0.760	103.0	31.7	0.122	20.0	3.2	0.593	207 950
**6-*star*-(PMA$_{150}$-*b*-PAS$_{200}$-N_3)**	6-*star*-(PMA$_{150}$-*b*-PAS$_{200}$)	272 992	1	0.003	0.8	184.0	0.540	73.0	29.4	0.086	14.0	3.2	0.680	272 517

Table S6. Details of **6-*star*-(PMA-*b*-PAS)-CNO** hybrids synthesis procedures.

Product	6-*star*-(PMA-*b*-PAS$_2$-N_3)				CNO (mg)	DMF (mL)	Product mass (g)
	Substrate	$M_{n. th}$ (g mol^{-1})	n (mmol)	m (g)			
**6-*star*-(PMA$_{25}$-*b*-PAS$_{200}$)-CNO**	6-*star*-(PMA$_{25}$-*b*-PAS$_{200}$-N_3)	207 950	0.003	0.560	10.0	10.0	0.465
**6-*star*-(PMA$_{150}$-*b*-PAS$_{200}$)-CNO**	6-*star*-(PMA$_{150}$-*b*-PAS$_{200}$-N_3)	272 517	0.002	0.620	10.0	10.0	0.550

Table S7. Details of the **6-*star*-(PMA-*b*-PVPh)-CNO** hybrids synthesis procedures.

Product	6-*star*-(PMA-*b*-PAS)-CNO		0.5 M KOH/MeOH (mL)	MeOH (mL)	Product mass (g)
	Substrate	m (g)			
**6-*star*-(PMA$_{25}$-*b*-PVPh$_{200}$)-CNO**	6-*star*-(PMA$_{25}$-*b*-PAS$_{200}$)-CNO	0.445	2.0	2.0	0.303
**6-*star*-(PMA$_{150}$-*b*-PVPh$_{200}$)-CNO**	6-*star*-(PMA$_{150}$-*b*-PAS$_{200}$)-CNO	0.530	2.0	2.0	0.407

Table S8. Details of **CP-5** and **CP-6** synthesis procedures.

**Product**	**6-*star*-(PMA-*b*-PVPh)**		**HCHO**		**10% NaOH (mL)**	**Product (g)**
			**1st portion**	**2nd portion**		
	**Substrate $M_{n,\ th}$ (g mol^{-1})**	**m (g)**	**V (mL)**	**V (mL)**		
**CP-5**	6-*star*-(PMA_{25}-*b*-$PVPh_{200}$)-CNO	0.285	0.161	0.645	2.14	1.14
**CP-6**	6-*star*-(PMA_{150}-*b*-$PVPh_{200}$)-CNO	0.385	0.155	0.618	2.55	1.07

Table S9. Summary of molecular weights and polydispersity indexes obtained from ^{1}H NMR and SEC analyses.

**Polymer**	**$M_{n,\ th}$** ^{a}	**$M_{n,\ NMR}$** ^{b}	**wt % PMA** ^{c}	**$M_{n,\ SEC}$** ^{d}	**Đ** ^{d}
**6-*star*-(PMA_{25})**	13 797	20 512	-	33 156	1.62
**6-*star*-(PMA_{50})**	26 710	36 525	-	65 329	1.66
**6-*star*-(PMA_{100})**	52 537	83 013	-	75 325	1.73
**6-*star*-(PMA_{150})**	78 364	104 191	-	131 368	1.73
**6-*star*-(PMA_{25}-*b*-PAS_{200})**	208 425	-	6.2	74 167	1.52
**6-*star*-(PMA_{50}-*b*-PAS_{200})**	221 338	-	13.4	124 171	1.43
**6-*star*-(PMA_{100}-*b*-PAS_{200})**	247 165	-	24.9	189 081	1.53
**6-*star*-(PMA_{150}-*b*-PAS_{200})**	272 992	-	36.2	215 766	1.58

^{a} $M_{n,\ th} = M_{CTA} + M_{MA} \cdot equiv_{MA}$ or $M_{n,\ th} = M_{CTA} + M_{MA} \cdot equiv_{MA} + M_{AS} \cdot equiv_{AS}$; the degree of monomer conversion was not considered. ^{b} $M_{n,\ NMR} = M_{CTA} + M_{MA} \cdot x_{MA} \cdot 6$ or $M_{n,\ NMR} = M_{CTA} + (M_{MA} \cdot x_{MA} + M_{AS} \cdot x_{AS}) \cdot 6 x_{MA}$ is integrated signal taken from ^{1}H NMR spectrum corresponding to singlet from 3H of $-CH_3$ group (PMA repeating unit) at 3.57 ppm compared to a quartet of $-CH_2-$ at 4.63 ppm. ^{c} wt % PMA = $(M_{MA} \cdot x_{MA})/(M_{MA} \cdot x_{MA} + M_{AS} \cdot x_{AS})$, where x_{MA} and x_{AS} are integrated signals taken from ^{1}H NMR spectrum; x_{MA} was defined by integrating singlet from 3H of $-CH_3$ group at 3.57 ppm, while x_{PAS} corresponds to integrated multiplet at approx. 6.2-6.6 ppm from 4 H of an aromatic ring. ^{d} Measured by SEC.

Table S10. Best-fit frequencies for the D and G bands obtained at 785 nm laser excitation energy and the relative I_D/I_G.

Sample	v_D (cm^{-1})	v_G (cm^{-1})	v_{2D} (cm^{-1})	I_D/I_G
**CNO**	1299	1596	2595	2.42
**6-*star*-(PMA$_{150}$-*b*-PVPh$_{200}$)-CNO**	1308	1612	2616	1.83
**CNO-SC(S)OEt**	1298	1601	2596	2.27
**CNO-PAS**	1304	1601	2598	1.84

Table S11. Surface Elemental Composition of selected carbon samples determined by XPS.

Sample	Elements (%)			
	C	O	N	Na
**C-1**	69.33	23.62	-	7.06
**C-4**	79.59	17.51	-	2.90
**C-5**	58.06	29.47	0.55	11.92
**C-6**	71.46	21.79	0.55	6.19
**C-7**	81.03	14.56	0.72	3.69

Table S12. Chemical state, positions, FWHM, and relative area percentages of the deconvoluted C 1s peaks obtained from XPS analyses of **C-1**, **C-4**, **C-5**, **C-6**, and **C-7** samples.

Region	Species	C-1			C-4			C-5			C-6			C-7		
		**Peak (eV)**	**FWHM (eV)**	**% Area**	**Peak (eV)**	**FWHM (eV)**	**% Area**	**Peak (eV)**	**FWHM (eV)**	**% Area**	**Peak (eV)**	**FWHM (eV)**	**% Area**	**Peak (eV)**	**FWHM (eV)**	**% Area**
**C 1s A**	C-H sp^3	284.87	0.77	10.1	284.89	0.78	10.2	284.97	0.78	9.6	284.96	0.78	9.4	284.87	0.77	11.9
**C 1s B**	C=C sp^2	284.33	0.81	52.9	284.35	0.80	46.9	284.37	0.82	40.5	284.39	0.81	46.9	284.32	0.81	50.6
**C 1s C**	C-C sp^3	285.51	0.79	8.5	285.52	0.81	9.0	285.55	0.80	9.1	285.54	0.80	8.6	285.53	0.80	9.9
**C 1s D**	C-OH, C-N (aziridine)	286.28	0.89	7.7	286.30	0.89	9.1	286.23	0.89	8.6	286.20	0.89	9.1	286.23	0.89	7.8
**C 1s E**	C-O-C	287.03	0.78	4.0	287.06	0.78	5.4	287.04	0.86	6.5	287.03	0.86	5.5	286.98	0.78	3.9
**C 1s F**	C=O	287.71	0.79	3.2	287.74	0.80	4.9	287.78	0.79	4.1	287.82	0.79	4.0	287.69	0.79	2.8
**C 1s G**	O=C-O-	288.44	0.83	3.1	288.45	0.83	4.2	288.56	0.89	4.4	288.66	0.89	3.5	288.44	0.83	2.1
**C 1s H**	O=C-OH	289.35	1.01	2.9	289.35	1.00	4.3	289.65	1.02	8.3	289.64	1.02	3.7	289.28	1.01	2.8
**C 1s I**	π-π^*	290.28	1.21	3.4	290.62	1.22	2.4	290.62	1.22	3.4	290.85	1.43	4.5	290.29	1.21	3.2
**C 1s DCS**	defects in carbon structure	283.63	0.68	4.3	283.67	0.68	3.7	283.75	0.92	5.5	283.75	0.78	4.8	283.63	0.68	5.0

Table S13. Chemical state, positions, FWHM, and relative area percentages of the deconvoluted O 1s peaks obtained from XPS analyses of **C-1**, **C-4**, **C-5**, **C-6**, and **C-7** samples.

Region	Species	C-1			C-4			C-5			C-6			C-7		
		Peak (eV)	FWHM (eV)	% Area	Peak (eV)	FWHM (eV)	% Area	Peak (eV)	FWHM (eV)	% Area	Peak (eV)	FWHM (eV)	% Area	Peak (eV)	FWHM (eV)	% Area
**O 1s A**	C=O	531.15	1.39	26.1	530.85	1.39	21.2	531.34	1.34	37.8	531.29	1.39	13.6	531.05	1.39	12.4
**O 1s B**	C-OH/epoxy	532.16	1.42	40.6	531.99	1.42	29.3	532.01	1.37	37.6	532.17	1.42	39.2	532.14	1.42	48.5
**O 1s C**	C-O-C	533.16	1.40	20.1	533.04	1.41	33.3	533.03	1.35	17.8	533.05	1.41	31.6	533.10	1.40	24.6
**O 1s D**	Ph-OH, O=C-O-	534.24	1.44	13.2	534.24	1.44	16.3	533.93	1.39	6.8	534.02	1.44	15.6	534.07	1.44	14.6

Table S14. Chemical state, positions, FWHM, and relative area percentages of the deconvoluted N 1s peaks obtained from XPS analyses of **C-6.**

Region	Species	Peak (eV)	FWHM (eV)	% Area
**N 1s A**	pyridinic-N	398.25	1.57	18.4
**N 1s B**	aziridine	399.83	1.57	51.5
**N 1s C**	quaternary N	401.27	1.57	20.2
**N 1s D**	pyridinic-N oxide	403.30	1.57	9.9

Table S15. TG results for selected polymers and carbon materials.

Sample	Maxima of the degradation rates (°C)	Residue at 900 °C (%)
**6-*star*-(PMA_{150})**	410	1
**6-*star*-(PMA_{150}-*b*-PAS_{200})**	410	4
**CP-4**	420, 885	26
**C-4**	760	63
**CP-6**	420, 870	31
**CNO-SC(S)OEt**	460	98
**CNO-PAS**	410	7
**CP-7**	190, 380, 430, 760	17

Table S16. Specific capacitances of the polymer or hybrid materials after pyrolysis, calculated from the CV studies.

Sample	C_S from integration of i_c vs. E from -600 to 0 mV, vs. Ag/AgCl ($F\ g^{-1}$)	C_S from slope of i_c vs. *sweep rate* relation ($F\ g^{-1}$)
**C-1**	19	22
**C-2**	28	-
**C-3**	38	-
**C-4**	50	56
**C-5**	86	109
**C-6**	139	132
**C-7**	68	-

Table S17. Specific capacitances of **C-6** for the different supporting electrolyte calculated from the CV studies.

**Supporting electrolyte**	**C_S from integration of i_c vs. E voltammogram from -600 to 0 mV, vs. Ag/AgCl (F g^{-1})**
**$TBAPF_6$**	139
**TBA acetate**	70
**$TBABF_4$**	103
**TBAP**	104
**$TEAPF_6$**	111
